# Supplementary material for: Rewiring of endogenous signaling pathways to genomic targets for therapeutic cell reprogramming
Source: Nat Commun. 2020 Jan 30;11:608. doi: 10.1038/s41467-020-14397-8 (PMC6992713; doi:10.1038/s41467-020-14397-8)
Supplement: Supplementary file 1 — Supplementary Information [file 41467_2020_14397_MOESM1_ESM.pdf]

Supplementary Information

Rewiring of endogenous signaling pathways to genomic targets for therapeutic cell reprogramming

Krzysztof Krawczyk[1], Leo Scheller[1], Hyojin Kim[1] and Martin Fussenegger[1,2,*]

[1]Department of Biosystems Science and Engineering, ETH Zurich, Mattenstrasse 26, CH-4058 Basel, Switzerland.

[2]Faculty of Science, University of Basel, Mattenstrasse 26, CH-4058 Basel, Switzerland

*Corresponding author. E-mail: fussenegger@bsse.ethz.ch

Table of contents:

Supplementary Figure 1. Nuclear translocation $GEAR_{NFAT}$

Supplementary Figure 2. Kinetics of $GEAR_{NFAT}$ -mediated transgene expression

Supplementary Figure 3. Endogenous insulin expression

Supplementary Figure 4. GEAR-mediated transgene expression in immortalized human mesenchymal stem cells

Supplementary Figure 5. Synthetic receptors MESA

Supplementary Figure 6. Unspecific effect of $GEAR_{NFAT}$ expression on endogenous gene transcription in Jurkat cells.

Supplementary Figure 7. $GEAR_{NFAT}$ plasmid titration

Supplementary Figure 8. Calcium-inducible dCas9-based nuclear translocation protein "CaRROT"

Supplementary Figure 9. Negative control experiments – transgene expression

Supplementary Figure 10. Negative control experiments – endogenous gene expression

Supplementary Figure 11. Effect of GEAR expression on the basal transcription of endogenous genes

Supplementary Figure 12. Dose-response relationships

Supplementary Figure 13. SEAP assay and qPCR comparability

Supplementary Table 1. Plasmids used and designed in this study

Supplementary Table 2. Synthetic guide RNA sequences

Supplementary Table 3. Detailed transfection protocols

Supplementary Table 4. qPCR primer pairs

Supplementary Table 5. Detailed statistics

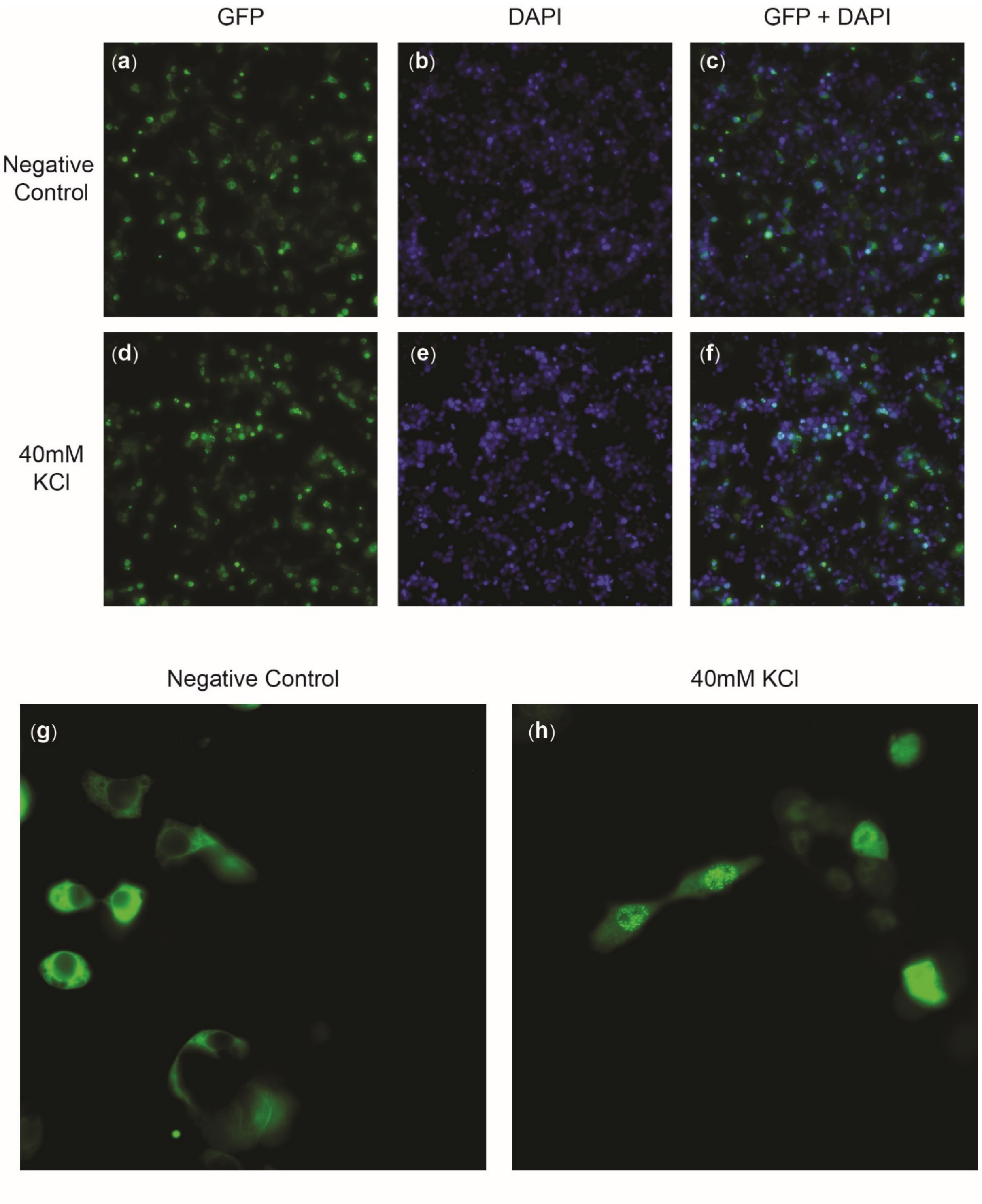
GFP
DAPI
GFP + DAPI
Negative Control
40mM KCl
(a)
(b)
(c)
(d)
(e)
(f)
Negative Control
40mM KCl
(g)
(h)

Supplementary Figure 1. Nuclear translocation of $\mathbf{GEAR_{NFAT}}$. β-Mimetic cells ($Ca_V1.3$-transgenic HEK293T) transfected with plasmids encoding $GEAR_{NFAT}$ were depolarized with 40 mM KCl for 4 hours. Images were captured with a wide-field fluorescence microscope. Panels **a**, **b**, **c** and **g** show non-induced cells (Negative Control). Panels **d**, **e**, **f**, and **h** show depolarized cells (40 mM KCl). Green fluorescence of GFP-tagged $GEAR_{NFAT}$ is presented in panels **a**, **d**, **g**, and **h**, while panels **b** and **e** show blue fluorescence of DAPI-stained nuclei. Panels **c** and **f** present merged channels for non-induced and depolarized cells, respectively. Panels **a**-**f** were captured using a 10x magnification objective. For panels **g** and **h**, a 40x magnification objective was used.

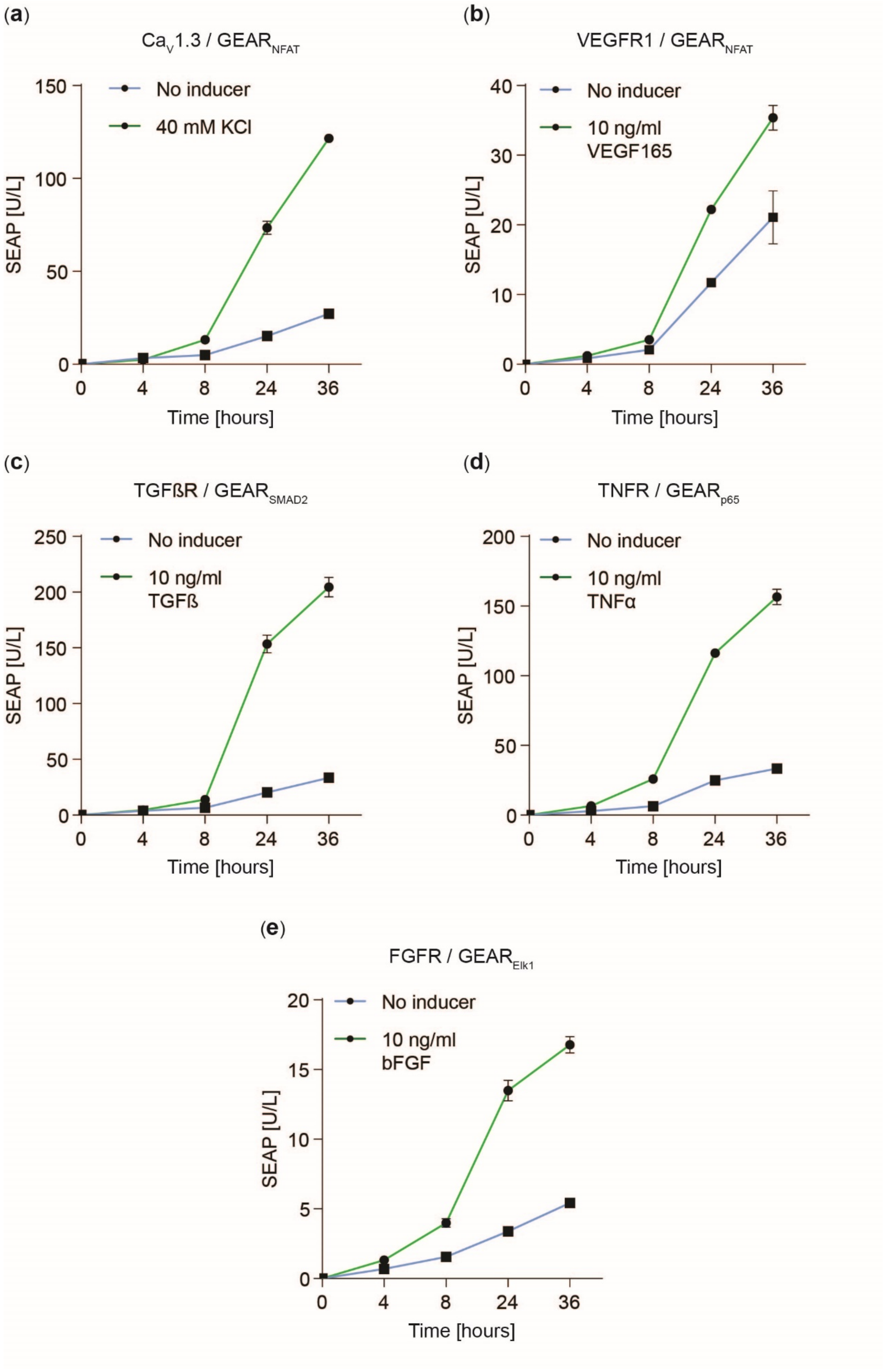

Supplementary Figure 2. Kinetics of GEAR-mediated transgene expression. Cells were transfected with plasmids encoding human insulin promoter-specific sgRNA ($sgRNA_{INS}$),

SEAP reporter plasmid controlled by human insulin promoter (P_{hINS}-SEAP), dCas9, an indicated GEAR and an indicated receptor. At 48 hours post-transfection, cells were stimulated with a compound indicated in the panel and SEAP was quantified from the supernatant after 4, 8, 24 and 36 hours. **(a)** Membrane depolarization (40 mM KCl) of β-mimetic cells (Ca_V1.3-transgenic HEK293T) and $GEAR_{NFAT}$ activation. (**b**) 10 ng/ml VEGF165-mediated $GEAR_{NFAT}$ activation. (**c**) 10 ng/ml TGFβ-mediated $GEAR_{SMAD2}$ activation. (**d**) 10 ng/ml TNFα-mediated $GEAR_{p65}$ activation. (**e**) 10 ng/ml bFGF-mediated $GEAR_{Elk1}$ activation. Numbers above the data points indicate fold induction in the stimulated cells (green line) compared to the negative control (blue line). Black dots correspond to mean. Error bars show the standard error of the mean (SEM). n = 3 biological replicates. Source data are provided as a Source Data file.

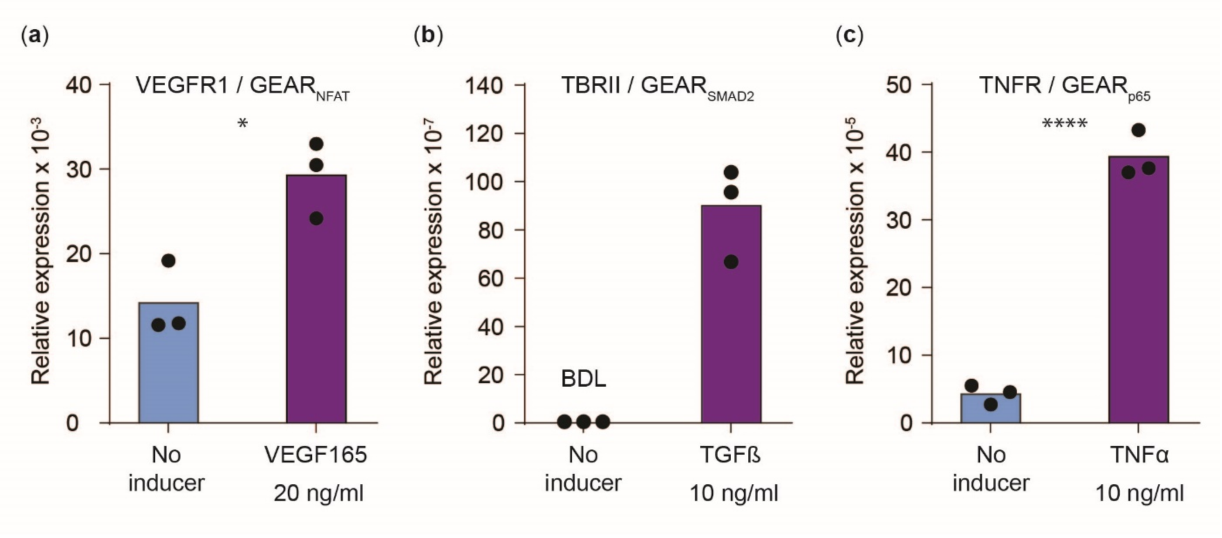

Supplementary Figure 3. Endogenous insulin expression. HEK293T cells expressing dCas9 and P_{hINS}-specific sgRNA ($sgRNA_{INS}$) were stimulated for 36 h with the indicated inducers. Insulin mRNA levels were quantified in relation to GAPDH. (**a**) VEGF165-induced $GEAR_{NFAT}$. (**b**) TGFβ-induced $GEAR_{SMAD2}$. (**c**) TNFα-induced $GEAR_{p65}$. Violet bars represent mRNA expression (relative to GAPDH) in stimulated cells (mean value). Blue bars represent mRNA expression in controls without inducer. Black dots correspond to individual data points of n = 3 biological replicates. BDL – below detection limit. * $p<0.05$, **** $p<0.0001$. Statistical significance was calculated using a two-tailed t-test. A detailed description of the statistical analysis is provided in Supplementary Table 5. Source data are provided as a Source Data file.

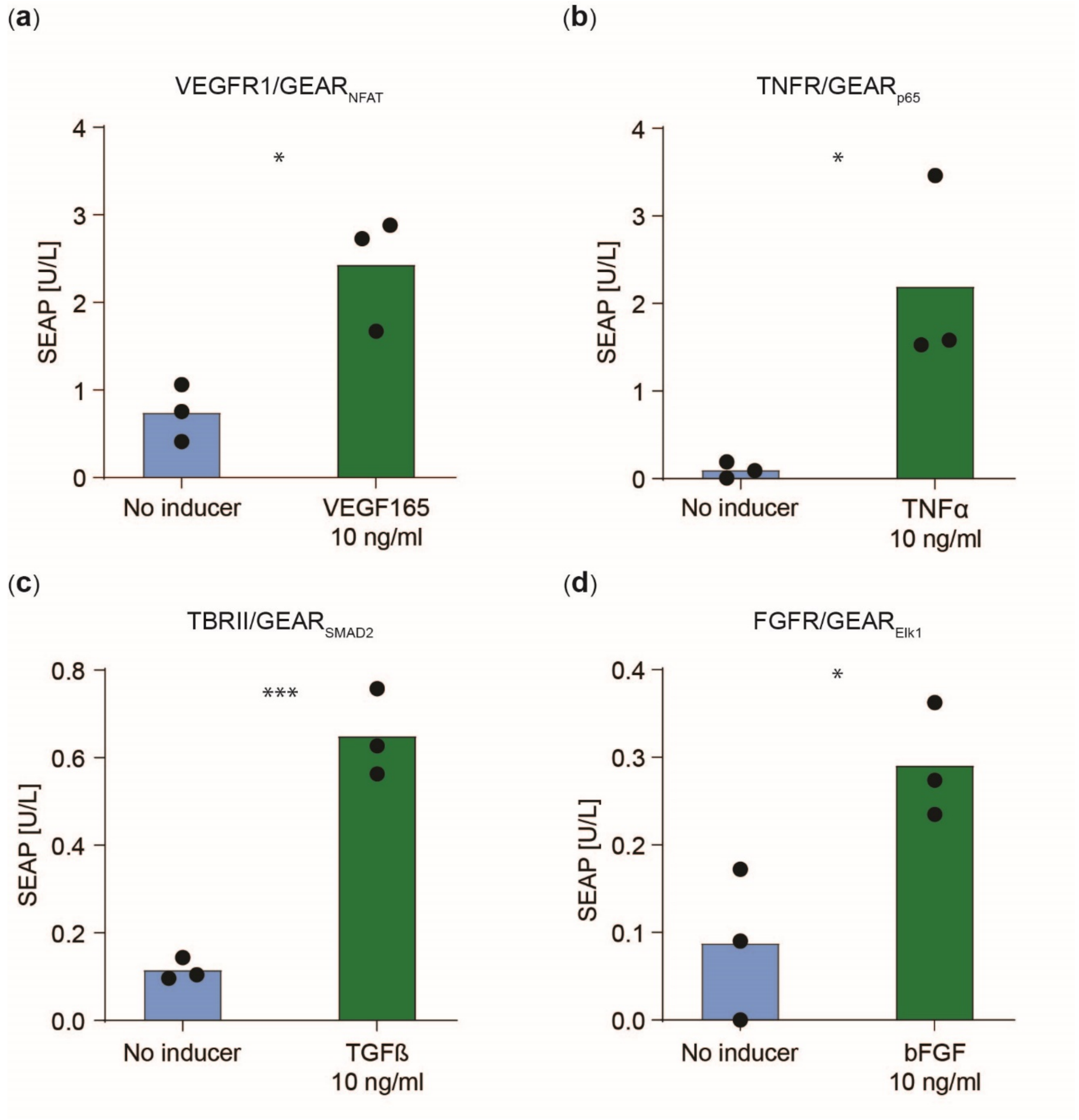

Supplementary Figure 4. GEAR-mediated transgene expression in immortalized human mesenchymal stem cells. hMSC-TERT cells containing a SEAP reporter plasmid under control of the human insulin promoter (P_{hINS}-SEAP) and expressing dCas9, as well as the indicated receptors and GEARs were stimulated for 36 hours with the indicated inducers. SEAP was quantified from the cell culture supernatant. (**a**) VEGF receptor 1 (VEGFR1) and $GEAR_{NFAT}$, (**b**) TNFα receptor (TNFR), $GEAR_{p65}$ and IκB, (**c**) TGFβ receptor II (TBRII) and $GEAR_{SMAD2}$, (**d**) endogenous FGF receptor (FGFR) and $GEAR_{Elk1}$. Black dots correspond to individual data points of n = 3 biological replicates. *p<0.05, ***p<0.001. Statistical significance was calculated using a two-tailed t-test. A detailed description of the statistical analysis is provided in Supplementary Table 5. Source data are provided as a Source Data file.

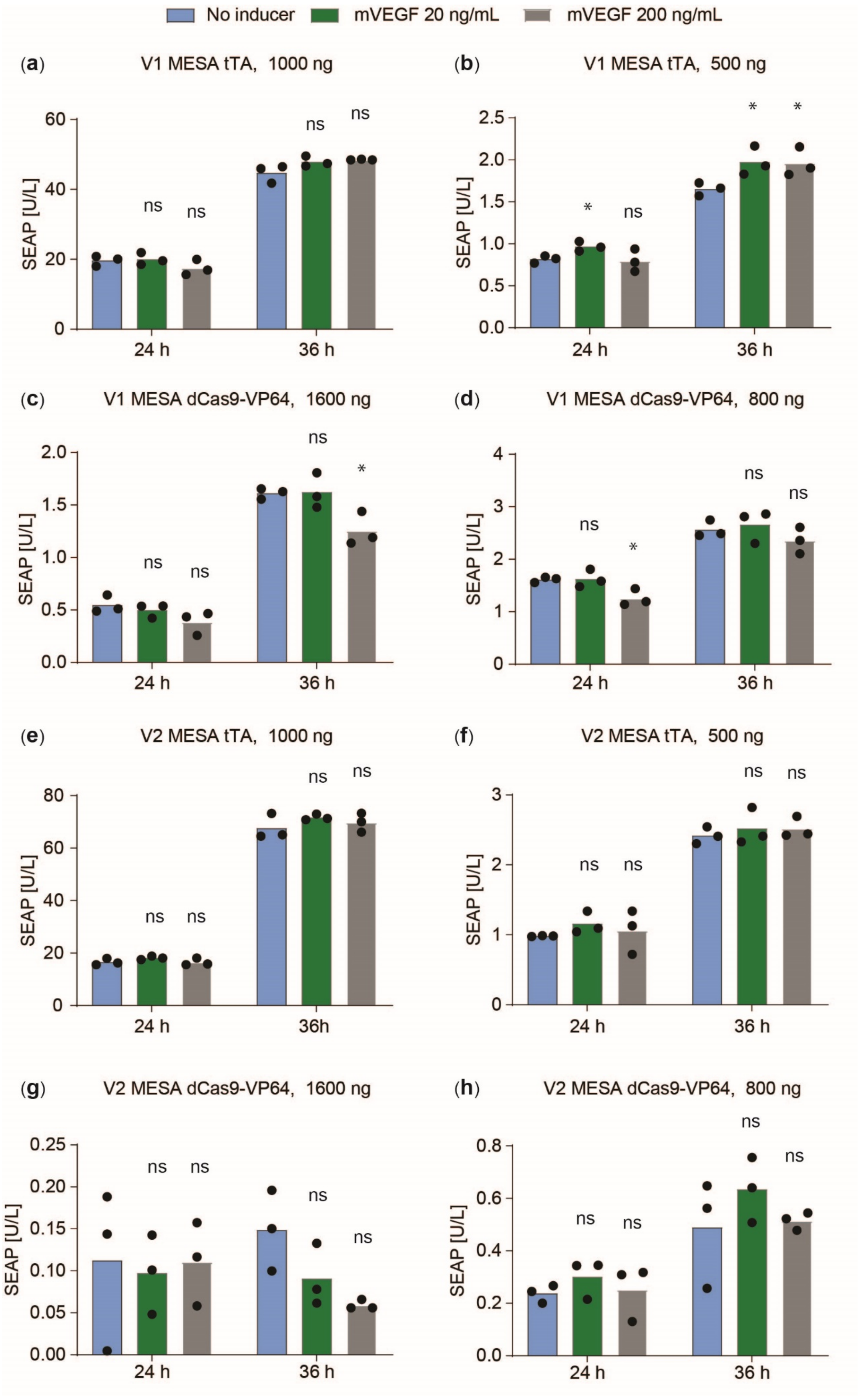

No inducer
mVEGF 20 ng/mL
mVEGF 200 ng/mL
(a)
V1 MESA tTA, 1000 ng
(b)
V1 MESA tTA, 500 ng
(c)
V1 MESA dCas9-VP64, 1600 ng
(d)
V1 MESA dCas9-VP64, 800 ng
(e)
V2 MESA tTA, 1000 ng
(f)
V2 MESA tTA, 500 ng
(g)
V2 MESA dCas9-VP64, 1600 ng
(h)
V2 MESA dCas9-VP64, 800 ng
SEAP [U/L]
24 h
36 h
36h
ns
*

Supplementary Figure 5. Synthetic receptors, MESA. HEK293T cells were transfected with plasmids encoding a human insulin promoter-specific sgRNA ($sgRNA_{INS}$), and (**a,b**) a SEAP reporter plasmid controlled by tetracycline repressor binding sites (O_{TetR}-$P_{hCMVmin}$-SEAP), plasmids for V1 MESA tTA and V1 MESA TEV, (**c,d**) a SEAP reporter plasmid controlled by the human insulin promoter (P_{hINS}-SEAP), and plasmids for V1 MESA dCas9-VP64 and V1 MESA TEV, (**e,f**) a SEAP reporter plasmid controlled by tetracycline repressor binding sites (O_{TetR}-$P_{hCMVmin}$-SEAP), and plasmids for V2 MESA tTA and V2 MESA TEV, (**g,h**) a SEAP reporter plasmid controlled by human insulin promoter (P_{hINS}-SEAP), and plasmids for V2 MESA dCas9-VP64 and V2 MESA TEV. Cells were induced with the indicated concentrations of recombinant mouse VEGF-164 (mVEGF). At 36 hours after stimulation, SEAP was quantified in the supernatant. Values above the graphs indicate the total amount of two MESA-encoding plasmids per 1 well of a 24-well plate. These plasmids were transfected at a ratio of MESA-transactivator (tTA or dCas9) to MESA-TEV of 24:1, as described by Schwartz *et al.*[1] Black dots correspond to individual data points of n = 3 biological replicates. ns – nonsignificant, *$p<0.05$. Statistical significance was calculated using a two-tailed t-test. A detailed description of the statistical analysis is provided in Supplementary Table 5. Source data are provided as a Source Data file.

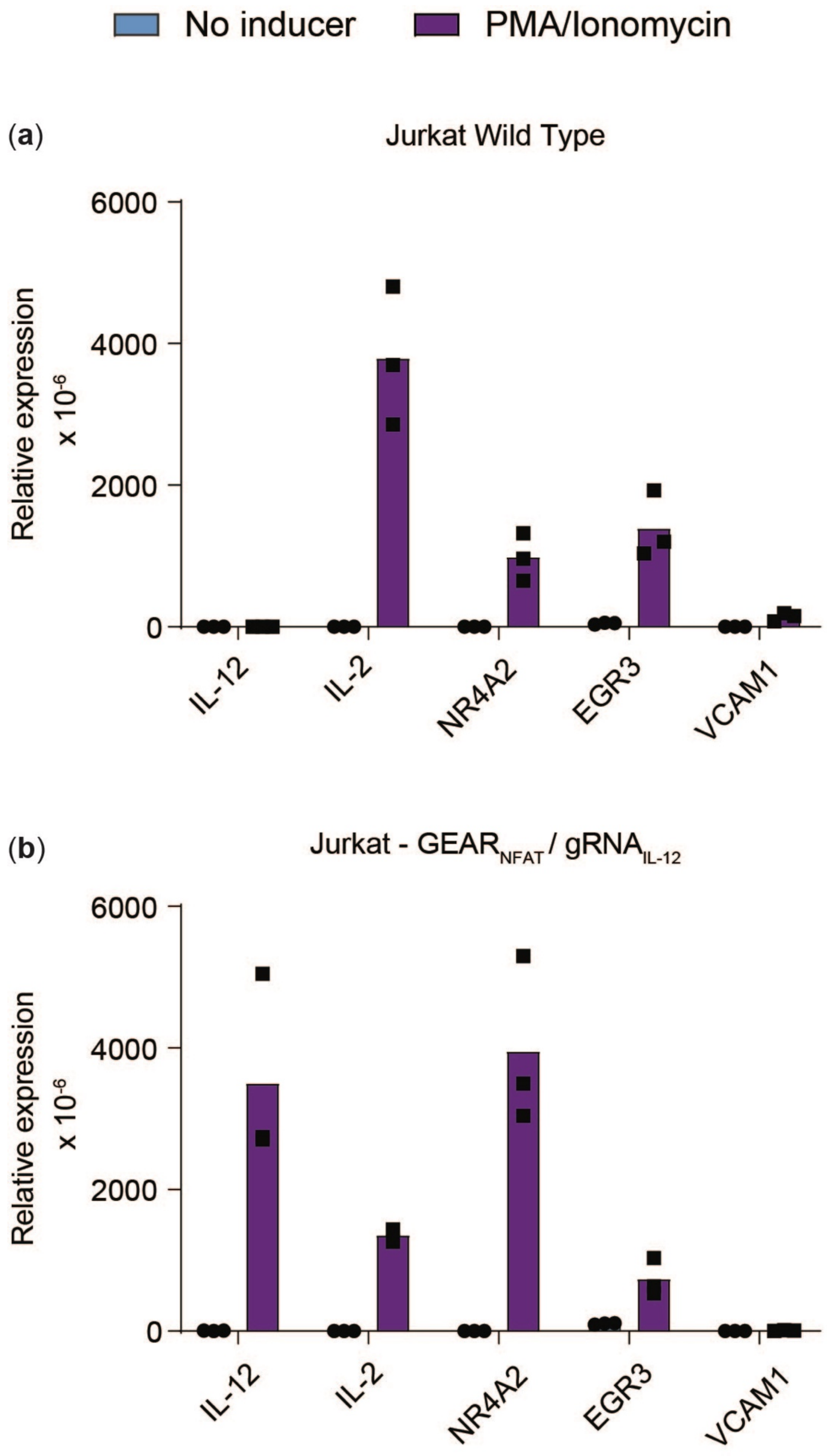

Supplementary Figure 6. Unspecific effect of GEAR$_{NFAT}$ expression on endogenous gene transcription in Jurkat cells. (**a**) Wild-type Jurkat cells, or (**b**) Jurkat cells stably expressing

dCas9, $GEAR_{NFAT}$, and interleukin 12B promoter-specific sgRNA ($sgRNA_{IL-12}$) were stimulated for 8 hours with 0.5 µg/mL ionomycin and 5 ng/mL phorbol 12-myristate 13-acetate (PMA). mRNA levels were quantified relative to GAPDH. Bars represent mean values for stimulated cells (violet bars) and control with no inducer (blue bars). Black dots correspond to individual data points of n = 3 biological replicates. Source data are provided as a Source Data file.

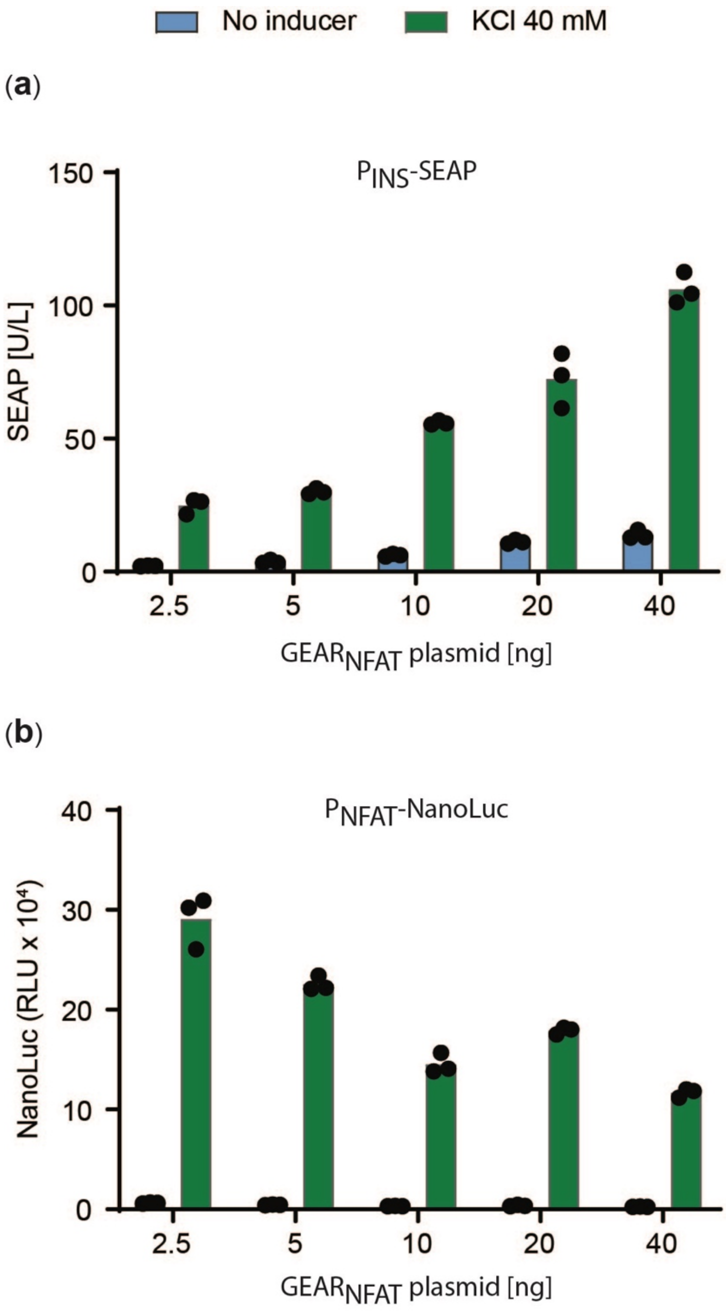

Supplementary Figure 7. GEAR$_{NFAT}$ plasmid titration. β-Mimetic cells (Ca$_V$1.3-transgenic HEK293T) were transfected with plasmids encoding dCas9 and the indicated amounts of GEAR$_{NFAT}$, human insulin promoter-specific sgRNA (sgRNA$_{INS}$), as well as the SEAP reporter plasmid controlled by human insulin promoter (P$_{hINS}$-SEAP), and the NanoLuc reporter plasmid controlled by an NFAT-dependent promoter (P$_{NFAT}$-NanoLuc). Cells were depolarized with 40 mM KCl. (**a**) SEAP and (**b**) NanoLuc were quantified from the cell culture supernatant after 48 hours. Black dots correspond to individual data points of n = 3 biological replicates. Source data are provided as a Source Data file.

HEK293T / Ca_V1.3 / CaRROT

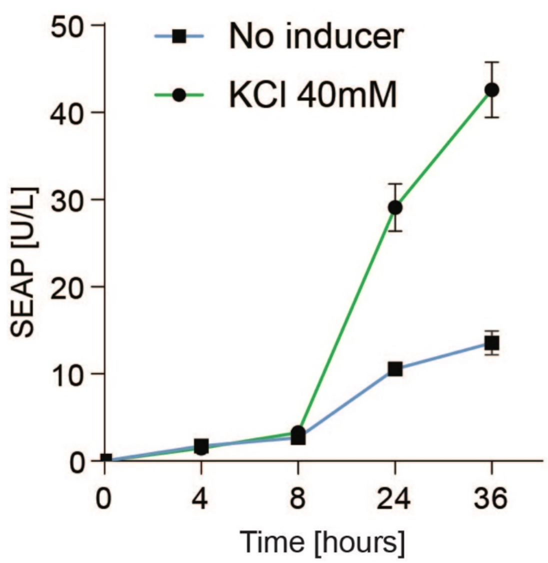

Supplementary Figure 8. Calcium-inducible dCas9-based nuclear translocation protein "CaRROT". (a) β-Mimetic cells (Ca_V1.3-transgenic HEK293T) were transfected with plasmids encoding a human insulin promoter-specific sgRNA ($sgRNA_{INS}$), as well as the SEAP reporter plasmid controlled by the human insulin promoter (P_{hINS}-SEAP) and CaRROT. At 48 hours post-transfection cells were depolarized with 40 mM KCl and SEAP was quantified from supernatant samples after 4, 8, 24 and 36 hours. Black dots correspond to mean. Error bars show standard error of the mean (SEM). n = 3 biological replicates. Source data are provided as a Source Data file.

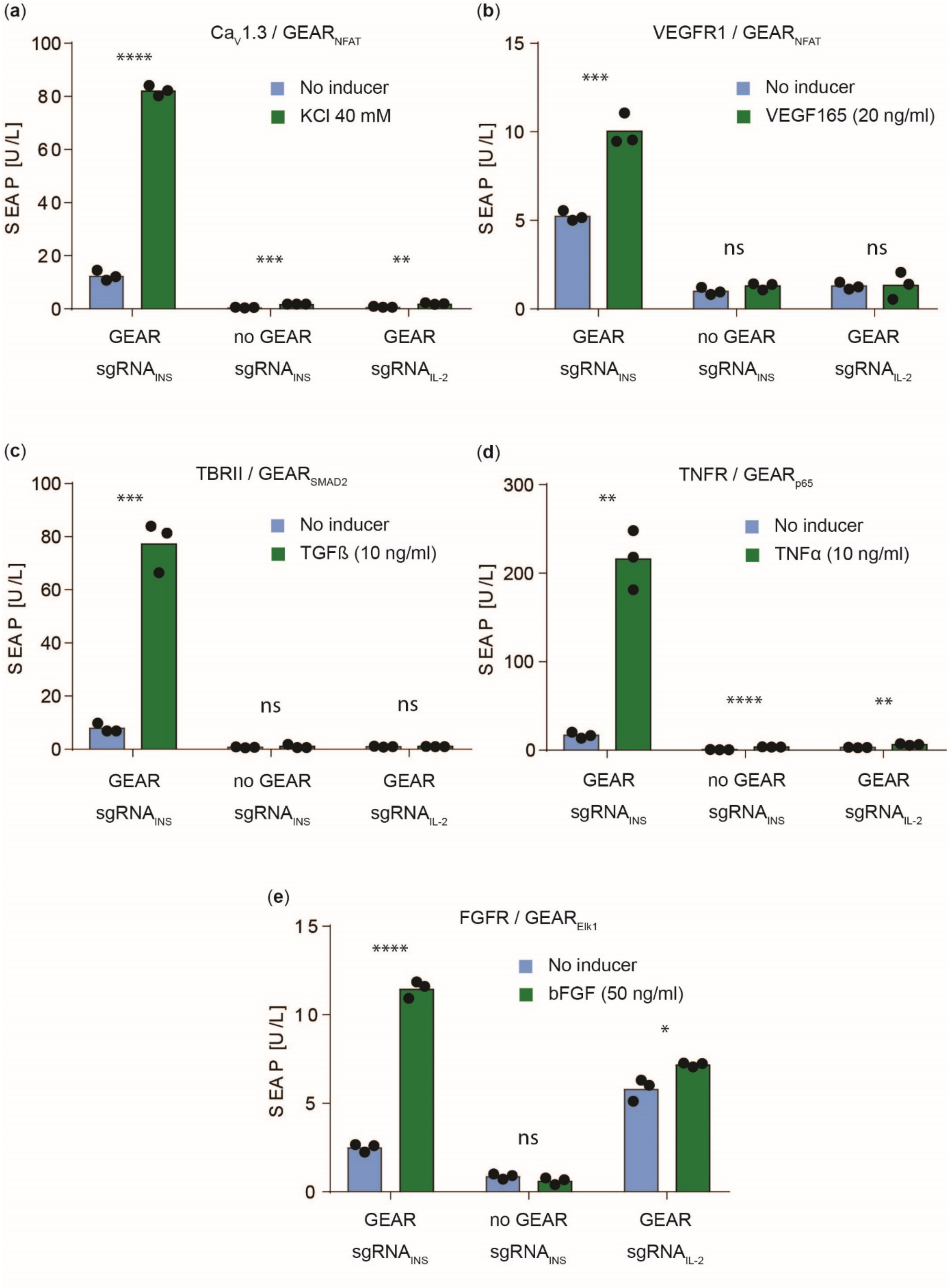
(a)
CaV1.3 / GEARNFAT
SEAP [U/L]
100
80
60
40
20
0
****
***
**
No inducer
KCl 40 mM
GEAR
sgRNAINS
no GEAR
sgRNAINS
GEAR
sgRNAIL-2
(b)
VEGFR1 / GEARNFAT
SEAP [U/L]
15
10
5
0
***
ns
ns
No inducer
VEGF165 (20 ng/ml)
GEAR
sgRNAINS
no GEAR
sgRNAINS
GEAR
sgRNAIL-2
(c)
TBRII / GEARSMAD2
SEAP [U/L]
100
80
60
40
20
0
***
ns
ns
No inducer
TGFß (10 ng/ml)
GEAR
sgRNAINS
no GEAR
sgRNAINS
GEAR
sgRNAIL-2
(d)
TNFR / GEARp65
SEAP [U/L]
300
200
100
0
**
****
**
No inducer
TNFα (10 ng/ml)
GEAR
sgRNAINS
no GEAR
sgRNAINS
GEAR
sgRNAIL-2
(e)
FGFR / GEARElk1
SEAP [U/L]
15
10
5
0
****
ns
*
No inducer
bFGF (50 ng/ml)
GEAR
sgRNAINS
no GEAR
sgRNAINS
GEAR
sgRNAIL-2

Supplementary Figure 9. Negative control experiments – transgene expression. At 24 hours after the beginning of transfection, HEK293T cells containing a SEAP reporter plasmid for human insulin promoter (P_{hINS}-SEAP) and expressing dCas9 were stimulated for 36 hours with the indicated inducers in the presence or absence of GEARs, as well as in the presence or absence of P_{hINS}-specific sgRNA ($sgRNA_{INS}$). SEAP was quantified from cell culture supernatant. In No-GEAR control cells, GEARS were replaced by an equal amount of a plasmid expressing MCP-YPet under control of the identical promoter (YPet is a yellow fluorescent protein). sgRNA-negative control cells were transfected with a plasmid encoding a sgRNA targeting human interleukin 2 promoter ($sgRNA_{IL-2}$). Green bars represent SEAP concentrations measured in the supernatant of stimulated cells (mean values). Blue bars represent controls without inducer. Black dots correspond to individual data points of n = 3 biologically independent samples. (**a**) Membrane depolarization-induced $GEAR_{NFAT}$. (**b**) VEGF165-induced $GEAR_{NFAT}$. (**c**) TGFβ-induced $GEAR_{SMAD2}$. (**d**) TNFα-induced $GEAR_{p65}$. (**e**) bFGF-induced $GEAR_{Elk1}$. ns – nonsignificant, *p<0.05, ** p<0.01, *** p<0.001, **** p<0.0001. Statistical significance was calculated using a two-tailed t-test. A detailed description of the statistical analysis is provided in Supplementary Table 5. Source data are provided as a Source Data file.

(a)

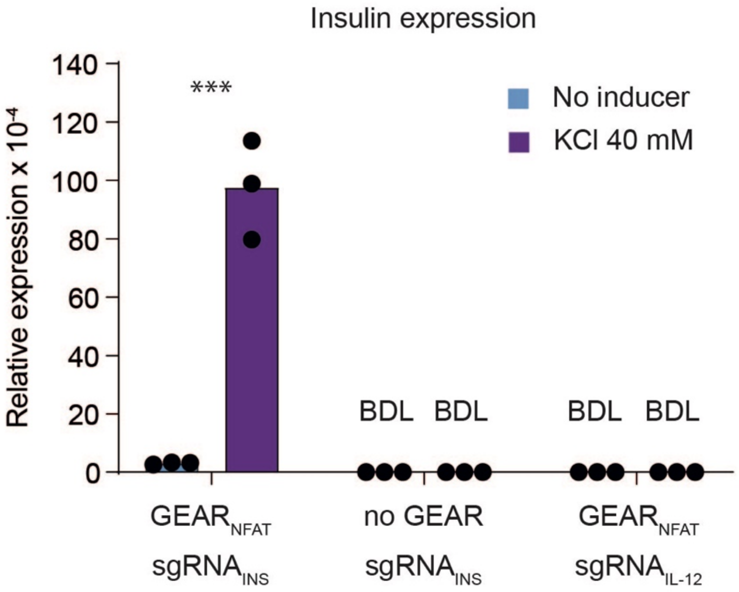

(b)

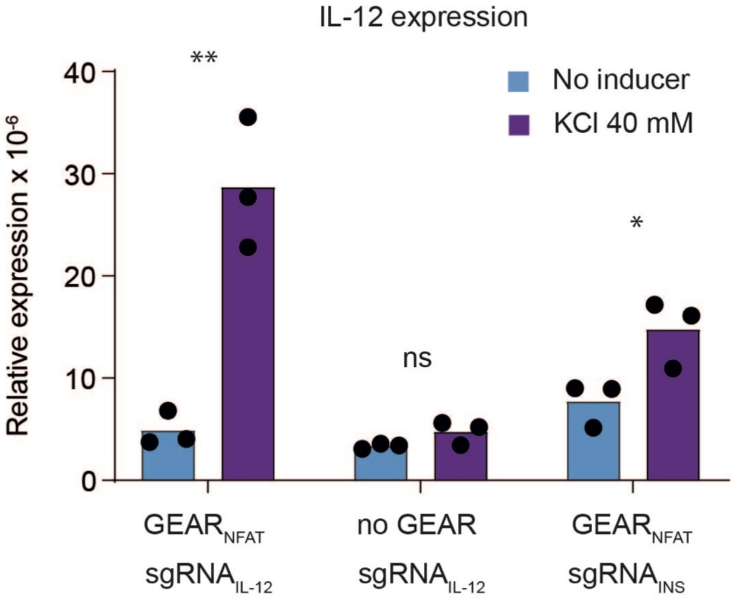

Supplementary Figure 10. Negative control experiments – endogenous gene expression. At 24 hours after the beginning of transfection, HEK293T cells expressing dCas9 were stimulated for 36 hours with the indicated inducers in the presence or absence of GEARs, as well as in the presence or absence of specific sgRNAs. mRNA levels were quantified in relation to GAPDH. In No-GEAR control cells, GEARS were replaced by an equal amount of a plasmid expressing MCP-YPet under control of the identical promoter. Blue bars represent relative gene expression for controls without inducer (mean values). Violet bars represent relative gene expression for stimulated cells (mean values). Black dots correspond to individual data points

of n = 3 biologically independent samples. (**a**) Endogenous insulin expression. (**b**) Endogenous interleukin 12 (IL-12) expression. BDL – below detection limit. ns – nonsignificant. *p<0.05, ** p<0.01, *** p<0.001. Statistical significance was calculated using a two-tailed t-test. A detailed description of the statistical analysis is provided in Supplementary Table 5. Source data are provided as a Source Data file.

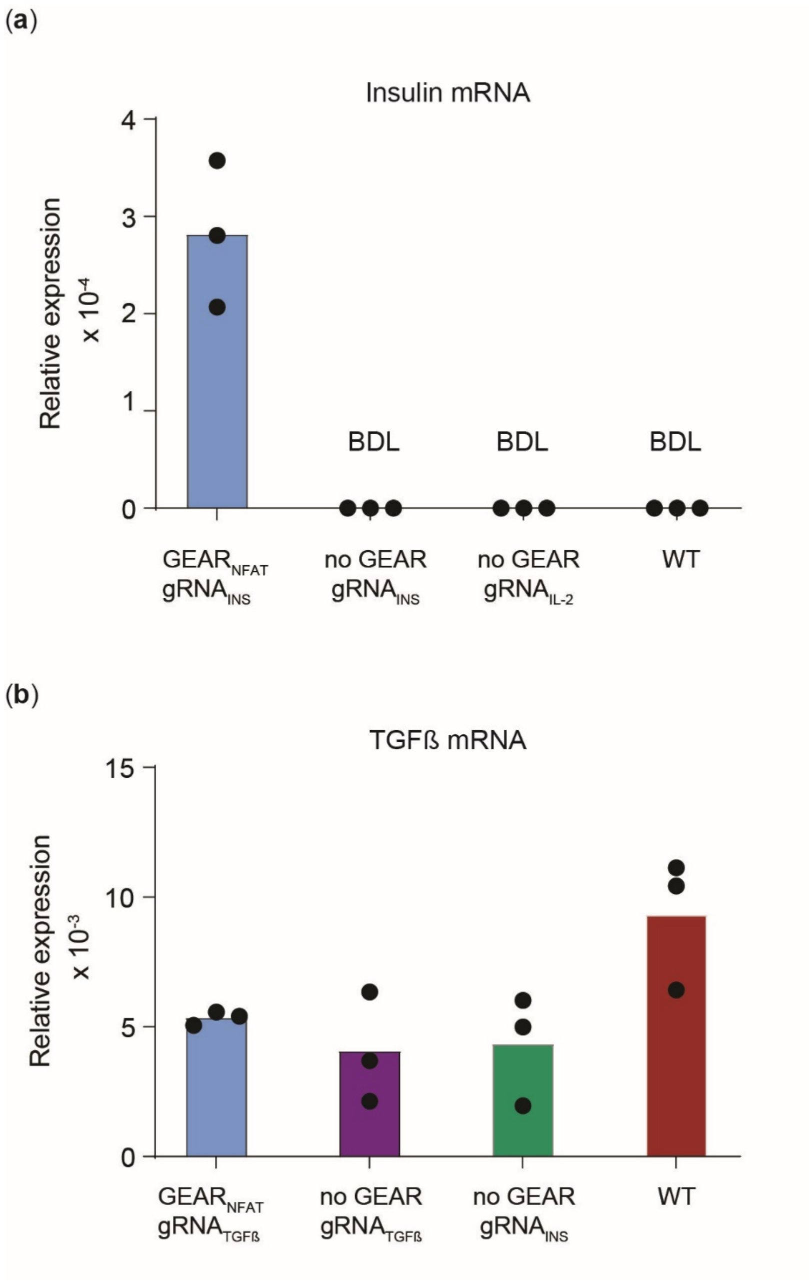

Supplementary Figure 11. Effect of GEAR expression on basal transcription of endogenous genes. HEK293T cells transfected with plasmids encoding dCas9, the indicated sgRNAs, the indicated GEARs, or not transfected (WT). RNA was isolated and (**a**) insulin mRNA, or (**b**) TGFβ mRNA was quantified. Plasmid encoding a fusion protein MCP-YPet (P_{hCMV}-MCP-YPet) was used for the "no GEAR" controls. BDL – below detection limit. Black dots correspond to individual data points of n = 3 biological replicates. Source data are provided as a Source Data file.

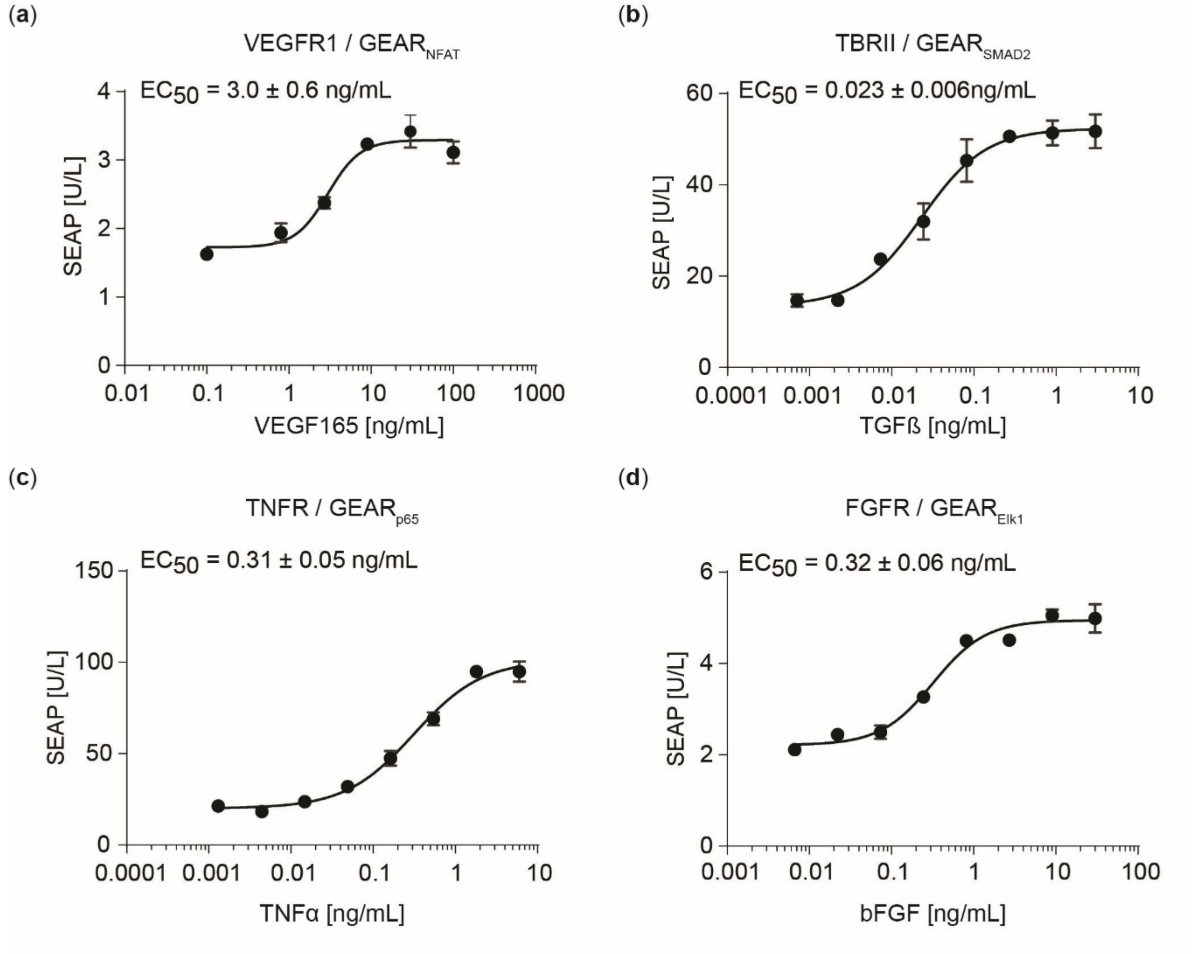

Supplementary Figure 12. Dose-response relationships. HEK293T cells containing a reporter plasmid for human insulin promoter (P_{hINS}-SEAP) and expressing dCas9, human insulin promoter specific sgRNA ($sgRNA_{INS}$), as well as the indicated receptors and GEARs: (**a**) VEGF receptor 1 (VEGFR1) and $GEAR_{NFAT}$, (**b**) TGFβ receptor type II (TBRII) and $GEAR_{SMAD2}$ (**c**) TNFα receptor (TNFR), $GEAR_{p65}$ and IκB, (**d**) endogenous FGF receptor (FGFR) and $GEAR_{Elk1}$. Cells were stimulated for 24 hours with the indicated concentration of the inducer, and the reporter protein SEAP was quantified from the cell culture supernatant. Black dots represent mean. Error bars show standard error of the mean (SEM). n = 3 biological replicates. Solid lines represent four-parameter nonlinear fitted dose-response curves. EC_{50} – half maximal effective concentration ± 95% confidence range. Source data are provided as a Source Data file.

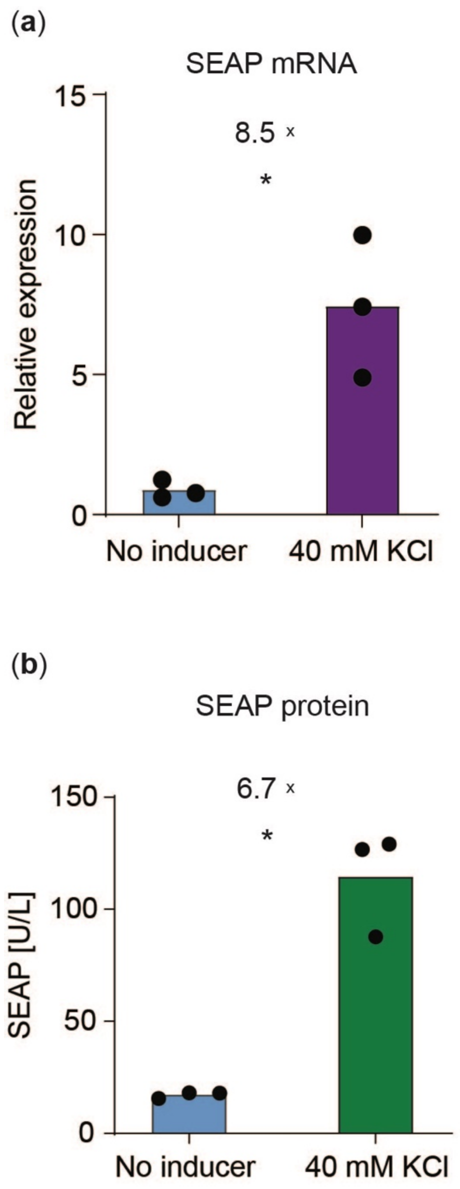

Supplementary Figure 13. SEAP assay and qPCR comparability. β-Mimetic cells expressing dCas9, GEAR$_{NFAT}$, a human insulin promoter-specific sgRNA (sgRNA$_{INS}$), as well as a SEAP reporter plasmid controlled by the human insulin promoter (P$_{hINS}$-SEAP) were depolarized with 40 mM KCl. 24 hours later (**a**) SEAP mRNA was quantified in the cell lysate and (**b**) SEAP protein was quantified in supernatant samples. 8.5 × and 6.7 × correspond to the fold induction values in stimulated cells (40 mM KCl) compared with noninduced cells. Black dots correspond to individual data points of n = 3 biological replicates. * $p<0.05$. Statistical significance was calculated using a two-tailed t-test. A detailed description of the statistical analysis is provided in Supplementary Table 5. Source data are provided as a Source Data file.

Supplementary Table 1. Plasmids used and designed in this study

Plasmid	Description and cloning strategy	Reference
pCaRROT-V5	Constitutive, P_{hCMV}-driven vector expressing calcium-inducible dCas9-based nuclear translocation protein "CaRROT" (P_{hCMV}-CaRROT-V5-pA).	[2]
pCaV1.3	Constitutive α_1D subunit of the murine L-type voltage-gated calcium channel $Ca_V1.3$ expression vector (P_{hCMV}-α_1D-pA) (Addgene no. 26576).	[3]
pCaVb3	Constitutive β_3 subunit of the murine L-type voltage-gated calcium channel expression vector (P_{hCMV}-β_3-pA) (Addgene no. 26574).	Lipscombe Lab (unpublished)
pCaVα2δ1	Constitutive α_2/δ_1 subunits the murine L-type voltage-gated calcium channel expression vector (P_{hCMV}-α_2/δ_1-pA) (Addgene no. 26575).	[4]
pcDNA3.1(+)	Constitutive P_{hCMV}-driven mammalian expression vector (P_{hCMV}-MCS-pA)	Thermo Fisher Scientific, CA
pCEP4YPet-MAMM	Constitutive YPet expression vector (P_{hCMV}-YPet-pA) (Addgene no. 14032).	[5]
pCGN-ELK-1	Constitutive human Elk-1 expression vector (P_{hCMV}-Elk1-pA) (Addgene no. 27156).	[6]
pCMV-T7-SB100	Constitutive SB100X expression vector (P_{hCMV}-SB100X-pA). (Addgene no. 34879).	[7]

Plasmid	Description and cloning strategy	Reference
pCOLADuet-1	Prokaryotic expression vector used as a filler plasmid for cotransfection of mammalian cells.	Merck, Germany
pdCas9-humanized	Catalytically dead, human codon-optimized Cas9 under the control of Murine Stem Cell retroVirus LTR promoter (Addgene no. 44246).	8
pFOX8	Constitutive YPet expression vector (P_{hCMV}-YPet-pA).	Unpublished
pGM47	Constitutive *Streptococcus pyogenes* catalytically dead CRISPR associated protein 9 (dCas9) expression vector (P_{hCMV}-dCas9 -pA). dCas9 was PCR-amplified from pdCas9-humanized using OMM223 (5‘-gc*gaattc*accatgactagtGACAAGAAGTATTCTATCGGAC-3‘) and OMM224 (5‘-aagctttctagacaccggt*ggatcc*gctagcAGCTCCCTCATCCCCTCCGAGCTG-3‘), digested with EcoRI/BamHI, and cloned into corresponding sites of pMM1 (GenBank accession code MN811115).	This work
pGM70	P_{hU6}-driven sgRNA complementary to a sequence in the human insulin promoter (P_{hU6}-sgRNA$_{hINS}$). OGM99 (5’-CACCGCGGCAGATGGCTGGGGGCTG-3’) and OGM100 (5’-AAACCAGCCCCCAGCCATCTGCCGC-3’) were annealed and cloned into BbsI-digested psgRNA(MS2) (GenBank accession code MN811116).	This work
pGM74	Constitutive *Streptococcus pyogenes* catalytically dead CRISPR associated protein 9 (dCas9) expression vector (P_{hCMV}-dCas9-NLS-pA). dCas9 was cloned from pGM47 (EcoRI/NheI) into pMM587 (EcoRI/SpeI) (GenBank accession code MN811114).	This work

Plasmid	Description and cloning strategy	Reference
pHA-NFAT1(4-460)-GFP	Constitutive expression of the regulatory domain of mouse NFAT1 (amino acids 4-460) (P_{hCMV}-HA-NFAT1(4-460)-GFP-pA) (Addgene no. 11107).	[9]
phFLT1	Constitutive expression of human VEGFR1 (P_{hCMV}-VEGFR1-pA) (Addgene no. 83435).	[10]
pHY57	P_{NFAT}-driven Fc-stabilized shGLP1 expression vector (P_{NFAT}-shGLP1-Fc-pA).	[11]
pKK05	Constitutive Kir2.1 expression vector (PhCMV-Kir2.1-pA). Kir2.1 was excised from pGEMTEZ-Kir2.1 using EcoRI and cloned into the corresponding site (EcoRI) of pcDNA3.1(+).	Unpublished
pKK110	Constitutive, P_{hCMV}-driven expression of MCP-YPet fusion protein (P_{hCMV}-MCP-YPet-pA). Ypet was cloned from pFOX8 (SpeI/BamHI) into pMM565 (NheI/BamHI) (GenBank accession code MN811103).	This work
pKK112	Constitutive, P_{SV40}-driven expression of a MCP-YPet fusion protein (P_{SV40}-MCP-YPet-pA). MCP-Ypet was cleaved from pKK110 using SpeI/BamHI and cloned into corresponding sites of pTS1016 (GenBank accession code MN811104).	This work
pKK122	Constitutive, P_{SV40}-driven $GEAR_{p65}$ expression (P_{SV40}-$GEAR_{p65}$-pA). Transcription factor p65 was PCR-amplified from HEK293T cDNA using OKK254 (5'-atta*actagt*ggtggtggaGACGAACTGTTCCCCCTC-3') and OKK255 (5'- atta*ggatcc*GCTGATCTGACTCAGCAGGG-3'), digested with SpeI/BamHI and cloned into NheI/BamHI-digested pKK112 (GenBank accession code MN811102).	This work
pKK126	P_{hU6}-driven sgRNA complementary to a sequence in the human interleukin 2 promoter. OKK248	This work

Plasmid	Description and cloning strategy	Reference
	(5’-*cacc*GAGGTAATGTTTTTCAGAC-3‘) and OKK249 (5’-*aaac*GTCTGAAAAACATTACCT-3‘) were annealed together and cloned into the BbsI sites of the plasmid psgRNA(MS2) (GenBank accession code MN811109).	
pKK130	Constitutive, P_{SV40}-driven IκB expression vector (P_{SV40}-IκB-pA). Human IκB was PCR-amplified from HEK293T cDNA using OKK266 (5’-atta*actagt*TTCCAGGCGGCCGAG-3’) and OKK267 (5’-atta*ggatccgctagc*TAACGTCAGACGCTGGCCTC-3’), digested with SpeI/BamHI, and cloned into the corresponding sites of pKK112 (GenBank accession code MN811112).	This work
pKK136	Constitutive, P_{hCMV}-driven $GEAR_{SMAD2}$ expression vector (P_{hCMV}-$GEAR_{SMAD2}$-pA). Human SMAD2 was PCR-amplified using OKK256 (5’- atta*actagt*ggtggtTCGTCCATCTTGCCATTC-3’) and OKK315 (5’-atta*tctaga*ctaTGACATGCTTGAGCAACG-3’), digested with SpeI/XbaI, and cloned into NheI/XbaI-digested pKK110 (GenBank accession code MN811108).	This work
pKK158	P_{hU6}-driven sgRNA complementary to a sequence in the human IL-12B promoter. OKK312 (5’-caccgAGTTTAAGTTTCCATCAGAA-3’) and OKK313 (5’-aaacTTCTGATGGAAACTTAAACTc-3’) were annealed and cloned into BbsI-digested psgRNA(MS2) (GenBank accession code MN811110).	This work
pKK160	P_{hU6}-driven sgRNA complementary to a sequence in the human TGFβ promoter. OKK329 (5’-caccgCCGCCCACGCGAGATGAGGA-3’) and OKK330 (5’-aaacTCCTCATCTCGCGTGGGCGGC-3’) were annealed and cloned into BbsI-digested psgRNA(MS2) (GenBank accession code MN811111).	This work

Plasmid	Description and cloning strategy	Reference
pKK171	ITR-containing vector for SB100X-specific transposon mediated stable genomic integrations, containing a constitutive dTomato and BlastR expression unit, and a constitutive expression unit for *Streptococcus pyogenes* catalytically dead CRISPR associated protein 9 (dCas9) (ITR-$P_{hEF1\alpha}$-dCas9-NLS-pA:P_{RPBSA}-dTomato-P2A-BlastR-pA-ITR). dCas9-NLS was PCR-amplified from pGM74 using OKK183 (5'-atta*ggcctgacaggcc*TTAGGGGTCCTCCACCTTGCGCTTCTTCTTGGGATCCCCTCCGAGCTGTG-3') and OKK184 (5'-atta*ggcctctgaggcc*ACCATGACTAGTGACAAGAAGTATTCTATCGG-3'), cleaved with SfiI and cloned into the corresponding site of pSBbi-RB (GenBank accession code MN811107).	This work
pKK172	ITR-containing vector for SB100X-specific transposon mediated stable genomic integrations, containing a constitutive dTomato and BlastR expression unit, and a constitutive expression unit for $GEAR_{NFAT}$ (ITR-$P_{hEF1\alpha}$-$GEAR_{NFAT}$-pA:P_{RPBSA}-dTomato-P2A-BlastR-pA-ITR). $GEAR_{NFAT}$ was PCR-amplified from pKK50 using OKK350 (5'-atta*ggcctctgaggcc*ACCATGGCTTCAAACTTTACTCAGTTCG-3') and OKK351 (5'-atta*ggcctgacaggcc*TTAAACGGGCCCTCTAGACTC-3'), cleaved with SfiI and cloned into corresponding site of pSBbi-RB (GenBank accession code MN811106).	This work
pKK44	Constitutive expression of a fusion protein that consists of MCP, the transactivation domain of transcription factor p65 ($p65_{TA}$), and the transactivation domain of heat shock factor 1 ($HSF1_{TA}$), without nuclear localization signal (P_{hCMV}-MCP-$p65_{TA}$-$HSF1_{TA}$-pA). MCP was PCR-amplified with OKK84 (5'-attaa*agctt*CGTACGGCCACCATGG-3') and OKK85 (5'-CGCTACCTCCTCCTCCGCTTC-3') from pMS2-P65-HSF1_Hygro. $p65_{TA}$-$HSF1_{TA}$ was PCR-amplified with OKK86 (5'-AAGCGGAGGAGGAGGTAGCGGACCTTCAGGGCAGATCAGC-3') and OKK87 (5'-	This work

Plasmid	Description and cloning strategy	Reference
	atta*gaattc*TGTACAGGAGACAGTGGGGTC-3'). The resulting fragments were assembled with a PCR reaction, amplified using OKK84 and OKK87, digested with HindIII/EcoRI, and cloned into corresponding sites of pcDNA3.1(+) (GenBank accession code MN811105).	
pKK50	Constitutive $GEAR_{NFAT}$ expression vector (P_{hCMV}-$GEAR_{NFAT}$-pA). MCP-$p65_{TA}$-$HSF1_{TA}$ was PCR-amplified from pKK44 using OKK84 (5'-atta*aagctt*CGTACGGCCACCATGG-3') and OKK96 (5'-CGCTGCCTCCTGAACCGCCGCTTCCGCCTGTACAGGAGACAGTGGGGTC-3'). $NFAT_{reg}$-GFP was PCR-amplified from pHA-NFAT1(4-460)-GFP using OKK97 (5'-GCGGTTCAGGAGGCAGCGGTGGATCAGGCTCCACCATGATCTTTTACCC-3') and OKK93 (5'-atta*gaattc*CTTGTACAGCTCGTCCATGC-3'). The resulting fragments were assembled by PCR to create MCP-$p65_{TA}$-$HSF1_{TA}$-$NFAT_{reg}$-GFP, were amplified using OKK84 and OKK93, and were cloned into pcDNA3.1(+) using HindIII/EcoRI (GenBank accession code MN811100).	This work
pKK82	Constitutive $GEAR_{Elk}$ expression vector. (P_{hCMV}-$GEAR_{Elk1}$-pA) MCP was PCR-amplified with OKK155 (5'-atta*acgcgtaagctt*CGTACGGCCACCATG-3') and OKK85 (5'-CGCTACCTCCTCCTCCGCTTC-3') from pKK44. Elk1 was PCR-amplified from pCGN-ELK-1 using OKK128 (5'-gaggaagcggaggaggaggtag*cggacc*GATCTCCCAGCCGCAG-3') and OKK129 (5'-ctagaaggcacagtcgaggctga*tctaga*TCATGGCTTCTGGGG-3'). The resulting fragments were assembled by PCR to create MCP-Elk1, were amplified using OKK155 and OKK129, and were cloned using HindIII/XbaI into pcDNA3.1(+) (GenBank accession code MN811101).	This work

Plasmid	Description and cloning strategy	Reference
pKK89	P_{NFAT}-driven secreted stabilized NanoLuc expression vector (P_{NFAT}–NanoLuc-Fc-pA). NanoLuc was PCR-amplified from pProinsulin-NanoLuc using OKK193 (5'-atta*gaattc*gccaccATGGGCGTCAAGGTGCTGTTCGCCCTCATTTGTATAGCTGTCGCTGAGGCGgtcttcacactcgaagatttcg-3') and OKK194 (5'-CCACTTCCACCGCCTCCCGCCAGAATGCGTTCG-3'). Fc fragment of a murine IgG was PCR-amplified from pHY57 using OKK191 (5'-GGAGGCGGTGGAAGTGGTGGTTGTAAGCCTTGCATATGTAC-'3') and OKK180 (5'-atta*gtcgac*CCACATTTGTAGAGGTTTTACTTGC-3'). The resulting fragments were assembled by PCR using OKK193 and OKK180, cleaved with EcoRI/SalI and cloned into corresponding sites of pMX57 (GenBank accession code MN811113).	This work
pLeo1207	ITR containing vector for SB100X-specific transposon mediated stable genomic integrations, containing a constitutive dTomato, PuroR expression unit and a constitutive expression unit for sgRNA complementary to a sequence in the human insulin promoter (ITR-P_{hU6}-$sgRNA_{hINS}$:P_{RPBSA}-BFP-P2A-PuroR-pA-ITR). P_{hU6}-$sgRNA_{hINS}$ was PCR-amplified from pGM70 using OKK356 (5'-atta*accggt*CGAGGGCCTATTTCCCATGATTCCTTC-3') and OKK357 (5'-atta*tctaga*CAGATGCGTAAGGAGAAAATACCGCATC-3'), cleaved with AgeI and XbaI and cloned into corresponding sites of pSBbi-BP (GenBank accession code MN811118).	This work
pLeo1209	ITR containing vector for SB100X-specific transposon mediated stable genomic integrations, containing a constitutive dTomato, PuroR expression unit and a constitutive expression unit for sgRNA complementary to a sequence in the human interleukin 12B promoter (ITR-P_{hU6}-$sgRNA_{hIL-12B}$:P_{RPBSA}-BFP-P2A-PuroR-pA-	This work

Plasmid	Description and cloning strategy	Reference
	ITR). P_{hU6}-$sgRNA_{hIL-12B}$ was PCR-amplified from pKK158 using OKK356 (5'-atta*accggt*CGAGGGCCTATTTCCCATGATTCCTTC-3') and OKK357 (5'-atta*tctaga*CAGATGCGTAAGGAGAAAATACCGCATC-3'), cleaved with AgeI and XbaI and cloned into corresponding sites of pSBbi-BP (GenBank accession code MN811119).	
pLeo628	Constitutive expression of $MAPK$-$GEMS_{RR120}$ (P_{SV40}-$MAPK$-$GEMS_{RR120}$ -pA)	[12]
pMF111	Mammalian reporter plasmid for TetR-Elk1 induced SEAP expression (O_{TetR}-$P_{hCMVmin}$-SEAP-pA)	[13]
pMM1	Cloning vector for mammalian gene expression (P_{hCMV}-MCS-pA).	[14]
pMM565	Constitutive expression of MCP (P_{hCMV}-MCP-pA).	[15]
pMM587	Constitutive expression of an NLS peptide (P_{hCMV}-NLS-pA).	Unpublished
pMS2-P65-HSF1_Hygro	Lentiviral vector containing a fusion protein consisting of MCP, a nuclear localization signal, the transactivation domain of transcription factor p65 ($p65_{TA}$), and the transactivation domain of heat shock factor 1 ($HSF1_{TA}$) ($P_{EF1\alpha}$-MCP-NLS-$p65_{TA}$- $HSF1_{TA}$-pA) (Addgene no. 61426).	[16]
pMX57	P_{NFAT}-driven SEAP expression vector (P_{NFAT}-SEAP-pA)	[17]
pProinsulin-NanoLuc	Lentiviral vector for constitutive expression of Proinsulin-NanoLuc (P_{hCMV}-Proinsulin-NanoLuc-pA). (Addgene no. 62057).	[18]

Plasmid	Description and cloning strategy	Reference
pSBbi-BP	ITR-containing vector for SB100X-specific transposon-mediated stable genomic integrations, containing a constitutive BFP and PuroR expression unit, and a constitutive expression unit (ITR-$P_{hEF1\alpha}$-MCS-pA:P_{RPBSA}-BFP-P2A-PuroR-pA-ITR). (Addgene no. 60512)	[7]
pSBbi-RB	ITR-containing vector for SB100X-specific transposon-mediated stable genomic integrations, containing a constitutive dTomato and BlastR expression unit, and a constitutive expression unit (ITR-$P_{hEF1\alpha}$-MCS-pA:P_{RPBSA}-dTomato-P2A-BlastR-pA-ITR). (Addgene no. 60512)	[7]
psgRNA(MS2)	sgRNA cloning vector with MCP-binding loops at tetraloop and stemloop 2 (Addgene no. 61424).	[16]
pSP20	P_{hINS}-driven SEAP expression vector (P_{hINS}-SEAP-pA) (GenBank accession code MN811117).	This work
pTBRII	Constitutive TBRII expression vector (P_{hCMV}-TBRII-pA) (Addgene no. 16622).	[19]
pTS1016	Constitutive, P_{SV40}-driven SEAP expression (P_{SV40}-SEAP-pA).	Unpublished
pV1-MESA-35F-M-tTA	Constitutive, P_{hCMV}-driven vector expressing MESA target chain with V2-MESA ectodomain, 35 extracellular linkers, a flag tag, the methionine-containing cleavage sequence, and tTA (P_{hCMV}-MESA V2 tTA-pA). (Addgene no. 84502).	[1]
pV1-MESA-45F-M-dCas9	Constitutive, P_{hCMV}-driven vector expressing MESA target chain with V1-MESA ectodomain, 45 extracellular linkers, a flag tag, the methionine-containing cleavage sequence, and dCas9-VP64 (P_{hCMV}-MESA V1 dCas9-VP64-pA). (Addgene no. 84504).	[1]

Plasmid	Description and cloning strategy	Reference
pV1-MESA-45F-M-tTA	Constitutive, P_{hCMV}-driven vector expressing MESA target chain with V1-MESA ectodomain, 45 extracellular linkers, a flag tag, the methionine-containing cleavage sequence, and tTA (P_{hCMV}-MESA V1 tTA-pA). (Addgene no. 84500).	1
pV1-MESA-45F-Tev	Constitutive, P_{hCMV}-driven vector expressing MESA protease chain with V1-MESA ectodomain, 45 extracellular linkers, a flag tag, and Tev protease (P_{hCMV}-MESA V1 TEV-pA). (Addgene no. 84501).	1
pV2-MESA-35F-M-dCas9	Constitutive, P_{hCMV}-driven vector expressing MESA target chain with V2-MESA ectodomain, 35 extracellular linkers, a flag tag, the methionine-containing cleavage sequence, and dCas9-VP64 (P_{hCMV}-MESA V2 dCas9-VP64-pA). (Addgene no. 84506).	1
pV2-MESA-35F-Tev	Constitutive, P_{hCMV}-driven vector expressing MESA protease chain with V2-MESA ectodomain, 35 extracellular linkers, a flag tag, and Tev protease (P_{hCMV}-MESA V2 TEV-pA). (Addgene no. 84503).	1

Oligonucleotides: Restriction endonuclease-specific sites are shown in italics and annealing sequences are shown in capital letters.

Abbreviations: **α_1D**, α_1D subunit of the murine L-type voltage-gated calcium channel $Ca_v1.3$; **α_2/δ_1**, α_2 and δ_1 subunits of the murine L-type voltage-gated calcium channel; **β_3**, β_3 subunit of the murine L-type voltage-gated calcium channel; **BFP**, blue fluorescent protein; **BlastR**, blasticidin resistance gene; **$Ca_v1.3$**, member 3 of the Ca_v1 family of L-type voltage-gated calcium channels; **CaRROT**, calcium-responsive transcriptional reprogramming tool; **CRISPR**, clustered regularly interspaced short palindromic repeats; **dCas9**, catalytically inactive *Streptococcus pyogenes* CRISPR associated protein 9; **dTomato**, dimeric red fluorescent protein variant; Fc – Fc fragment of murine IgG antibody; **HEK293T**, human endothelial kidney 293 cell line with stably incorporated Simian virus large T antigen; **HSF1**, human heat shock factor 1; **$HSF1_{TA}$**, human heat shock factor 1 transactivation domain; **GEAR**, generalized engineered activation regulator; **$GEAR_{Elk}$**, GEAR containing the transactivation domain of human Elk1; **$GEAR_{NFAT}$**, GEAR

containing the regulatory domain of murine NFAT1; **GEAR$_{p65}$,** GEAR containing the human transcription factor p65; **GEAR$_{SMAD2}$**, GEAR containing the human SMAD2; MAPK-GEMS$_{RR120}$, generalized extracellular molecule sensor activating MAPK pathway specific to RR120; **GFP**, green fluorescent protein; **HygroR**, hygromycin resistance gene; **ITR**, inverted terminal repeats of SB100X; **IκB**, nuclear factor of kappa light polypeptide gene enhancer in B-cells inhibitor; **MAPK**, mitogen-activated protein kinase; **MCP**, MS2 bacteriophage coat protein; **MCS**, multiple cloning site; **MESA**, modular extracellular sensor; **NanoLuc**, *Oplophorus gracilirostris luciferase*; **NLS**, nuclear localization signal; **NFAT**, nuclear factor of activated T-cells; **NFAT$_{reg}$,** nuclear factor of activated T-cells regulatory domain; O$_{TetR}$, TetR-binding operator sequence; **p65**, human transcription factor p65; **p65$_{TA}$**, human transcription factor p65 transactivation domain; **pA,** polyadenylation signal; **PCR**, polymerase chain reaction; **P$_{hCMV}$**, human cytomegalovirus immediate early promoter; **P$_{hEF1\alpha}$**, human elongation factor 1 alpha promoter; **P$_{hINS}$**, human insulin promoter; **P$_{NFAT}$**, synthetic mammalian promoter containing five tandem repeats of a human IL-4 NFAT-binding site; **P$_{RPBSA}$**, constitutive synthetic mammalian promoter; **P$_{SV40}$,** simian virus 40 promoter; **PuroR**, puromycin resistance gene; **SB100X**, optimized Sleeping Beauty transposase; **SEAP**, human placental secreted alkaline phosphatase; **sgRNA**, synthetic guide RNA; **shGLP1**, short human glucagon-like peptide 1; **SMAD2**, mothers against decapentaplegic homolog 2; **TBRII,** transforming growth factor β type II receptor; **TEV**, tobacco etch virus nuclear-inclusion-a endopeptidase; **tTA**, tetracycline-controlled transactivator; **VEGFR1**, vascular endothelial growth factor receptor, type 1; **YPet**, yellow fluorescent protein variant.

Supplementary Table 2. Synthetic guide RNA target sequences

Plasmid	Target	Complementary sequence	Reference
pGM70, pLeo1207	Human insulin promoter	5'-CGGCAGATGGCTGGGGGCTG-3'	This work
pKK126	Human interleukin 2 (IL-2) promoter	5'-AGGTAATGTTTTTTCAGAC-3'	[20]
pKK158, pLeo1209	Human interleukin 12 B (IL-12B) promoter	5'- AGTTTAAGTTTCCATCAGAA-3'	This work
pKK160	Human TGFβ promoter	5'- CCGCCCACGCGAGATGAGGA-3'	This work

Supplementary Table 3. Detailed transfection protocols used for Figures 2, 3, 5 and Supplementary Figures 3 and 9.

The amount of DNA was calculated per 1 well of a 24-well plate.

Application	GEAR	sgRNA	Other plasmids	Total DNA
Membrane depolarization-induced transgene activation	pKK50<br>10 ng	pGM70<br>25 ng	pGM74 – 25 ng<br>pKK05 – 15 ng<br>pSP20 – 150 ng<br>pCaV1.3 – 30 ng<br>pCaVα2δ1 – 30 ng<br>pCaVb3 – 10 ng	300 ng
Membrane depolarization-induced endogenous insulin activation	pKK50<br>10 ng	pGM70<br>25 ng	pGM74 – 50 ng<br>pKK05 – 15 ng<br>pCOLADuet-1 – 130 ng<br>pCaV1.3 – 30 ng<br>pCaVα2δ1 – 30 ng<br>pCaVb3 – 10 ng	300 ng

Application	GEAR	sgRNA	Other plasmids	Total DNA
VEGF165-induced transgene activation	pKK50<br>2.5 ng	pGM70<br>25 ng	pGM74 – 50 ng<br>pSP20 – 150 ng<br>phFLT1 – 25 ng<br>pCOLADuet-1 – 47.5 ng	300 ng
VEGF165-induced endogenous gene activation	pKK50<br>6.25 ng	pGM70<br>25 ng	pGM74 – 50 ng<br>phFLT1 – 25 ng<br>pCOLADuet-1 – 193.75 ng	300 ng
TGFβ-induced transgene activation	pKK136<br>25 ng	pGM70<br>25 ng	pGM74 – 50 ng<br>pSP20 – 150 ng<br>pTBRII – 25 ng<br>pCOLADuet-1 – 25 g	300 ng
TGFβ-induced endogenous gene activation	pKK136<br>25 ng	pGM70 or pKK158<br>25 ng	pGM74 – 50 ng<br>pTBRII – 25 ng	300 ng

Application	GEAR	sgRNA	Other plasmids	Total DNA
			pCOLADuet-1 – 175 ng	
TNFα-induced transgene activation	pKK122<br>150 ng	pGM70<br>25 ng	pGM74 – 25 ng<br>pSP20 – 150 ng<br>pKK130 – 150 ng	500 ng
TNFα-induced endogenous gene activation	pKK122<br>200 ng	pGM70 or pKK158<br>25 ng	pGM74 – 50 ng<br>pKK130 – 200 ng	475 ng
bFGF-induced transgene activation	pKK82<br>25 ng	pGM70<br>25 ng	pGM74 – 50 ng<br>pSP20 – 150 ng<br>pCOLADuet-1 – 50 ng	300 ng
RR120-induced transgene activation	pKK82<br>25 ng	pGM70<br>25 ng	pGM74 – 25 ng<br>pSP20 – 150 ng<br>pLeo628 – 275 ng	500 ng

Supplementary Table 4. qPCR primer pairs

Target	Sequence	Reference
Human insulin	5‘-ATCAGAAGAGGCCATCAAGCA-3‘<br>5‘-TAGAGAGCTTCCACCAGGTGTGA-3‘	[21]
Human GAPDH	5‘-GTCTCCTCTGACTTCAACAGCG-3‘<br>5‘-ACCACCCTGTTGCTGTAGCCAA-3‘	Origene Technologies<br>(Cat. No. HP205798)
Human interleukin 12B (IL-12B)	5‘-GACATTCTGCGTTCAGGTCCAG-3‘<br>5‘-CATTTTTGCGGCAGATGACCGTG-3‘	Origene Technologies<br>(Cat. no. HP205923)
SEAP	5‘-ACAAACTGGGGCCTGAGATACC-3‘<br>5‘-CTGCACTCAAGCCAATGGTCTG-3‘	This work
Human TGFβ	5‘-TACCTGAACCCGTGTTGCTCTC-’3<br>5‘-GTTGCTGAGGTATCGCCAGGAA-3’	Origene Technologies<br>(Cat. no. HP200609)
Human interleukin 2 (IL-2)	5‘-AGAACTCAAACCTCTGGAGGAAG-’3<br>5‘-GCTGTCTCATCAGCATATTCACAC-’3	Origene Technologies<br>(Cat. no. HP200553)
Human nuclear receptor related-1 protein (NR4A2)	5‘-AAACTGCCCAGTGGACAAGCGT-’3<br>5‘-GCTCTTCGGTTTCGAGGGCAAA-’3	Origene Technologies<br>(Cat. no. HP209171)

**Target**	**Sequence**	**Reference**
Human early growth response 3 (EGR3)	5‘-GACTCGGTAGTCCATTACAATCAG-’3<br>5‘-AGTAGGTCACGGTCTTGTTGCC-’3	Origene Technologies<br>(Cat. no. HP207741)
Human vascular cell adhesion molecule 1 (VCAM1)	5‘-GATTCTGTGCCCACAGTAAGGC-’3<br>5‘-TGGTCACAGAGCCACCTTCTTG-’3	Origene Technologies<br>(Cat. no. HP230503)

Supplementary Table 5. Detailed statistics

Confidence level = 95%

**Figure**	**Description**	**Mean 1 (control)**	**Mean 2 (induced)**	**Fold induction**	**F test to compare variances (p-value)**	**Welch's correction applied (yes/no)**	**Two-tailed t-test (p-value)**
2b	Ca_V1.3, $GEAR_{NFAT}$;	3.71	33.5	9.0	0.0092	yes	**0.0063**
2d	VEGFR, $GEAR_{NFAT}$	6.54	13.27	2.0	0.090	no	**0.0035**
2f	TBRII, $GEAR_{SMAD2}$	7.71	50.76	6.6	0.33	no	**0.00014**
2h	TNFR, $GEAR_{p65}$	4.55	89.28	19.6	0.0067	yes	**0.0085**
2j	FGFR, $GEAR_{Elk1}$	2.10	9.54	4.5	0.69	no	**0.00000037**
2l	GEMS-MAPK (RR120), $GEAR_{Elk1}$	1.40	14.41	10.3	0.067	no	**0.0000057**
3b	Ca_V1.3, $GEAR_{NFAT}$ – Insulin mRNA	3.02	97.42	32.3	0.0010	yes	**0.011**

Figure	Description	Mean 1 (control)	Mean 2 (induced)	Fold induction	F test to compare variances (p-value)	Welch's correction applied (yes/no)	Two-tailed t-test (p-value)
3c	Cav1.3, $GEAR_{NFAT}$ - Insulin protein	0	0.26	N/A	N/A	yes	**0.0004**
3d	Insulin	6.77	234.5	34.6	0.0097	yes	**0.0027**
	IL-12	11.3	81.26	7.2	0.017	yes	**0.021**
4b	$gRNA_{IL\text{-}12}$	12.34	8546	692.5	0.00000065	yes	**0.032**
	$gRNA_{INS}$	2.87	3.17	1.1	0.16	no	**0.7314**
4c	2 h	18.37	25.7	1.4	0.21	no	**0.17**
	4 h	23.92	322.3	13.5	0.037	yes	**0.0008**
	8 h	20.93	2112	100.9	0.74	no	**0.00000000004**
5b	TBRII, $GEAR_{SMAD2}$	8.83	14.51	1.6	0.48	no	**0.0080**
5d	TNFR, $GEAR_{p65}$	20.04	193.8	9.7	0.044	yes	**0.020**
S2	8 h	0.84	3.002	3.6	0.020	yes	**0.0397**

**Figure**	**Description**	**Mean 1 (control)**	**Mean 2 (induced)**	**Fold induction**	**F test to compare variances (p-value)**	**Welch's correction applied (yes/no)**	**Two-tailed t-test (p-value)**
	24 h	6.80	30.16	4.4	0.08	no	**0.000006**
	36 h	9.731	49.26	5.1	0.0045	yes	**0.0019**
	48 h	48.2	117.8	2.4	0.8037	no	**0.0003**
S3a	VEGFR, $GEAR_{NFAT}$	14.2	29.23	2.1	0.95	no	**0.014**
S3b	TBRII, $GEAR_{SMAD2}$	0	87.31	N/A	N/A	yes	**0.018**
S3c	TNFR, $GEAR_{p65}$	4.30	39.3	9.2	0.29	no	**0.000082**
S4a	VEGFR/$GEAR_{NFAT}$	0.74	2.42	3.3	0.39	no	**0.0164**
S4b	TNFR/ $GEAR_{p65}$	0.098	2.19	22.4	0.014	yes	**0.0305**
S4c	TBRII/ $GEAR_{SMAD2}$	0.11	0.65	5.7	0.12	no	**0.0008**
S4d	FGFR/$GEAR_{Elk1}$	0.087	0.29	3.3	0.73	no	**0.0314**
S5a	MESA V1 TetR 24 h 20 ng	19.65	20	1.0	0.82	no	**0.825**
	MESA V1 TetR 36 h 20 ng	44.72	47.85	1.1	0.498	no	**0.073**

Figure	Description	Mean 1 (control)	Mean 2 (induced)	Fold induction	F test to compare variances (p-value)	Welch's correction applied (yes/no)	Two-tailed t-test (p-value)
	MESA V1 TetR 24 h 200 ng	19.65	17.51	0.9	0.59	no	**0.237**
	MESA V1 TetR 36 h 200 ng	44.72	48.5	1.1	0.0045	no	**0.063**
S5b	MESA V1 TetR 24 h 20 ng	0.82	0.97	1.2	0.72	no	**0.107**
	MESA V1 TetR 36 h 20 ng	1.65	1.98	1.2	0.33	no	**0.005**
	MESA V1 TetR 24 h 200 ng	0.82	0.80	1.0	0.19	no	**0.839**
	MESA V1 TetR 36 h 200 ng	1.65	1.96	1.2	0.34	no	**0.013**
S5c	MESA V1 dCas9 24 h 20 ng	0.55	0.50	0.9	0.79	no	0.579
	MESA V1 dCas9 36 h 20 ng	1.61	1.62	1.0	0.17	no	0.902
	MESA V1 dCas9 24 h 200 ng	0.55	0.39	0.7	0.70	no	0.115
	MESA V1 dCas9 36 h 200 ng	1.61	1.26	0.8	0.18	no	0.021
S5d	MESA V1 dCas9 24 h 20 ng	1.61	1.62	1.0	0.17	no	0.948
	MESA V1 dCas9 36 h 20 ng	2.56	2.66	1.0	0.43	no	0.569

Figure	Description	Mean 1 (control)	Mean 2 (induced)	Fold induction	F test to compare variances (p-value)	Welch's correction applied (yes/no)	Two-tailed t-test (p-value)
	MESA V1 dCas9 24 h 200 ng	1.61	1.26	0.8	0.18	no	0.021
	MESA V1 dCas9 36 h 200 ng	2.56	2.36	0.9	0.58	no	0.299
S5e	MESA V2 TetR 24 h 20 ng	16.6	18.14	1.1	0.45	no	0.133
	MESA V2 TetR 36 h 20 ng	67.64	71.71	1.1	0.11	no	0.232
	MESA V2 TetR 24 h 200 ng	16.6	16.49	1.0	0.89	no	0.924
	MESA V2 TetR 36 h 200 ng	67.64	69.8	1.0	0.70	no	0.569
S5f	MESA V2 TetR 24 h 20 ng	0.98	1.16	1.2	0.0032	yes	0.128
	MESA V2 TetR 36 h 20 ng	2.42	2.52	1.0	0.34	no	0.570
	MESA V2 TetR 24 h 200 ng	0.98	1.06	1.1	0.0008	no	0.681
	MESA V2 TetR 36 h 200 ng	2.42	2.52	1.0	0.79	no	0.405
S5g	MESA V2 dCas9 24 h 20 ng	0.11	0.097	0.9	0.39	no	0.819
	MESA V2 dCas9 36 h 20 ng	0.15	0.091	0.6	0.75	no	0.175

Figure	Description	Mean 1 (control)	Mean 2 (induced)	Fold induction	F test to compare variances (p-value)	Welch's correction applied (yes/no)	Two-tailed t-test (p-value)
	MESA V2 dCas9 24 h 200 ng	0.11	0.11	1.0	0.43	no	0.981
	MESA V2 dCas9 36 h 200 ng	0.15	0.059	0.4	0.028	yes	0.033
S5h	MESA V2 dCas9 24 h 20 ng	0.24	0.30	1.3	0.34	no	0.250
	MESA V2 dCas9 36 h 20 ng	0.49	0.63	1.3	0.54	no	0.353
	MESA V2 dCas9 24 h 200 ng	0.24	0.25	1.1	0.19	no	0.831
	MESA V2 dCas9 36 h 200 ng	0.49	0.52	1.1	0.05	no	0.839
S6a	IL-12	0.00	1.47	N/A	N/A	yes	**0.50**
	IL-2	1301.47	3948.24	3.0	0.46	no	**0.0033**
	NR4A2	0.83	642.07	770.7	0.32	no	**0.00000006**
	EGR3	85.77	1072.99	12.5	0.063	no	**0.00092**
	FOSb	0.87	2.75	3.2	0.24	no	**0.48**
	ATF3	401.20	322.81	0.8	0.51	no	**0.30**

Figure	Description	Mean 1 (control)	Mean 2 (induced)	Fold induction	F test to compare variances (p-value)	Welch's correction applied (yes/no)	Two-tailed t-test (p-value)
	VCAM1	0.00	342.29	N/A	N/A	yes	**0.037**
S6b	IL-12	3.76	226.73	60.3	0.0042	yes	**0.0073**
	IL-2	593.28	1012.55	1.7	0.18	no	**0.11**
	NR4A2	2.52	331.47	131.4	9.15E-05	yes	**0.027**
	EGR3	52.70	358.27	6.8	0.020	yes	**0.0007**
	FOSb	2.07	13.18	6.4	0.041	yes	**0.091**
	ATF3	203.23	456.59	2.2	0.066	no	**0.020**
	VCAM1	0.00	16.63	N/A	N/A	yes	**0.082**
S8a	$GEAR_{NFAT}$	1.389	23.63	11.0	0.018	yes	**0.0018**
	CaRROT	1.61	5.55	3.4	0.82	no	**0.00002**
S8b	24 h	1.36	2.25	1.7	0.050	no	**0.0021**
	48 h	2.19	5.31	2.4	0.058	no	**0.0010**
S9a	$GEAR_{NFAT}$	12.47	82.2	6.6	0.98	no	**0.0000015**
	No GEAR	0.52	1.80	3.5	0.077	no	**0.00014**

Figure	Description	Mean 1 (control)	Mean 2 (induced)	Fold induction	F test to compare variances (p-value)	Welch's correction applied (yes/no)	Two-tailed t-test (p-value)
	control						
	No gRNA control	0.73	1.98	2.7	0.67	no	**0.0032**
S9b	$GEAR_{NFAT}$	5.235	10.03	1.9	0.18	no	**0.0010**
	No GEAR control	1.00	1.29	1.3	0.92	no	**0.14**
	No gRNA control	1.29	1.34	1.0	0.13	no	**0.93**
S9c	$GEAR_{SMAD2}$	7.87	77.25	9.8	0.063	no	**0.00023**
	No GEAR control	0.71	1.02	1.4	0.11	no	**0.50**
	No gRNA control	0.94	1.00	1.1	0.15	no	**0.58**

Figure	Description	Mean 1 (control)	Mean 2 (induced)	Fold induction	F test to compare variances (p-value)	Welch's correction applied (yes/no)	Two-tailed t-test (p-value)
S9d	$GEAR_{p65}$	16.89	215.8	12.8	0.021	yes	**0.0087**
	No GEAR control	0.80	3.74	4.6	0.75	no	**0.000036**
	No gRNA control	3.16	6.42	2.0	0.22	no	**0.0049**
S9e	$GEAR_{Elk1}$	2.51	11.47	4.6	0.37	no	**0.0000082**
	No GEAR control	0.88	0.62	0.7	0.77	no	**0.14**
	No gRNA control	5.82	7.19	1.2	0.064	no	**0.019**
S10a	$GEAR_{NFAT}$	0.61	117.58	192.74	0.000050	yes	**0.00059**
	No GEAR control	N/A	N/A	N/A	N/A	N/A	N/A
	No gRNA	N/A	N/A	N/A	N/A	N/A	N/A

Figure	Description	Mean 1 (control)	Mean 2 (induced)	Fold induction	F test to compare variances (p-value)	Welch's correction applied (yes/no)	Two-tailed t-test (p-value)
	control						
S10b	$GEAR_{NFAT}$	4.88	26.68	5.87	0.13	no	**0.0034**
	No GEAR control	3.36	4.76	1.41	0.08	no	**0.11**
	No gRNA control	7.70	14.75	1.92	0.61	no	**0.038**
S13a	SEAP mRNA	0.88	7.43	8.5	0.0316	yes	**0.045**
S13b	SEAP protein	17.16	114.5	6.7	0.0070	yes	**0.018**

N/A – not applicable

Supplementary references:

1 Schwarz, K. A., Daringer, N. M., Dolberg, T. B. & Leonard, J. N. Rewiring human cellular input-output using modular extracellular sensors. *Nat. Chem. Biol.* **13**, 202-209 (2017).

2 Nguyen, N. T., He, L., Martinez-Moczygemba, M., Huang, Y. & Zhou, Y. Rewiring calcium signaling for precise transcriptional reprogramming. *ACS Synth. Biol.* **7**, 814-821 (2018).

3 Xu, W. & Lipscombe, D. Neuronal Ca(V)1.3alpha(1) L-type channels activate at relatively hyperpolarized membrane potentials and are incompletely inhibited by dihydropyridines. *J. Neurosci.* **21**, 5944-5951 (2001).

4 Lin, Y., McDonough, S. I. & Lipscombe, D. Alternative splicing in the voltage-sensing region of N-Type CaV2.2 channels modulates channel kinetics. *J. Neurophysiol.* **92**, 2820-2830 (2004).

5 Nguyen, A. W. & Daugherty, P. S. Evolutionary optimization of fluorescent proteins for intracellular FRET. *Nat. Biotechnol.* **23**, 355-360 (2005).

6 Lee, S. M., Vasishtha, M. & Prywes, R. Activation and repression of cellular immediate early genes by serum response factor cofactors. *J. Biol. Chem.* **285**, 22036-22049 (2010).

7 Kowarz, E., Loscher, D. & Marschalek, R. Optimized Sleeping Beauty transposons rapidly generate stable transgenic cell lines. *Biotechnol. J.* **10**, 647-653 (2015).

8 Qi, L. S. *et al.* Repurposing CRISPR as an RNA-guided platform for sequence-specific control of gene expression. *Cell* **152**, 1173-1183 (2013).

9 Aramburu, J. *et al.* Affinity-driven peptide selection of an NFAT inhibitor more selective than cyclosporin A. *Science* **285**, 2129-2133 (1999).

10 Raikwar, N. S., Liu, K. Z. & Thomas, C. P. N-terminal cleavage and release of the ectodomain of Flt1 is mediated via ADAM10 and ADAM 17 and regulated by VEGFR2 and the Flt1 intracellular domain. *PLoS One* **9**, e112794 (2014).

11 Ye, H., Daoud-El Baba, M., Peng, R. W. & Fussenegger, M. A synthetic optogenetic transcription device enhances blood-glucose homeostasis in mice. *Science* **332**, 1565-1568 (2011).

12 Scheller, L., Strittmatter, T., Fuchs, D., Bojar, D. & Fussenegger, M. Generalized extracellular molecule sensor platform for programming cellular behavior. *Nat. Chem. Biol.* **14**, 723-729 (2018).

13 Fussenegger, M. *et al.* Streptogramin-based gene regulation systems for mammalian cells. *Nat. Biotechnol.* **18**, 1203-1208 (2000).

14 Muller, M. *et al.* Designed cell consortia as fragrance-programmable analog-to-digital converters. *Nat. Chem. Biol.* **13**, 309-316 (2017).

15 Müller, M. *et al.* Designed cell consortia as fragrance-programmable analog-to-digital converters. *Nat. Chem. Biol.* **13**, 309 (2017).

16 Konermann, S. *et al.* Genome-scale transcriptional activation by an engineered CRISPR-Cas9 complex. *Nature* **517**, 583-588 (2015).

17 Xie, M. *et al.* beta-cell-mimetic designer cells provide closed-loop glycemic control. *Science* **354**, 1296-1301 (2016).

18 Burns, S. M. *et al.* High-throughput luminescent reporter of insulin secretion for discovering regulators of pancreatic beta-cell function. *Cell Metab.* **21**, 126-137 (2015).

19 Zhou, S. *et al.* Targeted deletion of Smad4 shows it is required for transforming growth factor beta and activin signaling in colorectal cancer cells. *Proc. Natl. Acad. Sci. U S A* **95**, 2412-2416 (1998).

20 Hsu, P. D. *et al.* DNA targeting specificity of RNA-guided Cas9 nucleases. *Nat. Biotechnol.* **31**, 827-832 (2013).

21 Gimenez, C. A. *et al.* CRISPR-on system for the activation of the endogenous human INS gene. *Gene Ther.* **23**, 543-547 (2016).
