# Supplementary material for: Rewiring of endogenous signaling pathways to genomic targets for therapeutic cell reprogramming
Source: Nat Commun. 2020 Jan 30;11:608. doi: 10.1038/s41467-020-14397-8 (PMC6992713; doi:10.1038/s41467-020-14397-8)
Supplement: Supplementary file 2 — Reporting Summary [file 41467_2020_14397_MOESM2_ESM.pdf]

nature research

Corresponding author(s): Martin Fussenegger

Last updated by author(s): Dec 7, 2019

Reporting Summary

Nature Research wishes to improve the reproducibility of the work that we publish. This form provides structure for consistency and transparency in reporting. For further information on Nature Research policies, see Authors & Referees and the Editorial Policy Checklist .

Statistics

For all statistical analyses, confirm that the following items are present in the figure legend, table legend, main text, or Methods section.

n/a	Confirmed	
☐	☒	The exact sample size (*n*) for each experimental group/condition, given as a discrete number and unit of measurement
☐	☒	A statement on whether measurements were taken from distinct samples or whether the same sample was measured repeatedly
☐	☒	The statistical test(s) used AND whether they are one- or two-sided<br>*Only common tests should be described solely by name; describe more complex techniques in the Methods section.*
☐	☒	A description of all covariates tested
☐	☒	A description of any assumptions or corrections, such as tests of normality and adjustment for multiple comparisons
☐	☒	A full description of the statistical parameters including central tendency (e.g. means) or other basic estimates (e.g. regression coefficient) AND variation (e.g. standard deviation) or associated estimates of uncertainty (e.g. confidence intervals)
☐	☒	For null hypothesis testing, the test statistic (e.g. *F*, *t*, *r*) with confidence intervals, effect sizes, degrees of freedom and *P* value noted<br>*Give P values as exact values whenever suitable.*
☒	☐	For Bayesian analysis, information on the choice of priors and Markov chain Monte Carlo settings
☒	☐	For hierarchical and complex designs, identification of the appropriate level for tests and full reporting of outcomes
☒	☐	Estimates of effect sizes (e.g. Cohen's *d*, Pearson's *r*), indicating how they were calculated

Our web collection on statistics for biologists contains articles on many of the points above.

Software and code

Policy information about availability of computer code

Data collection	Absorbance and luminescence data was collected using Tecan Magellan version 4.00/4,0,0,31.<br>qPCR data was collected using Eppendorf Realplex version 2.2. Microscopic images were collected using Nikon Ti-U wide-field inverted microscope equipped with a Nikon Intensilight. Green fluorescence was recorded using a 469/35 nm excitation filter, 495 nm long pass filter, and 520/35 nm emission filter.
Data analysis	Calculations were performed using Microsoft Excel 2013. Statistical analysis was performed and graphs were made using GraphPad Prism version 7.04. Images were processed using ImageJ.

For manuscripts utilizing custom algorithms or software that are central to the research but not yet described in published literature, software must be made available to editors/reviewers. We strongly encourage code deposition in a community repository (e.g. GitHub). See the Nature Research guidelines for submitting code & software for further information.

Data

Policy information about availability of data

All manuscripts must include a data availability statement. This statement should provide the following information, where applicable:

- Accession codes, unique identifiers, or web links for publicly available datasets
- A list of figures that have associated raw data
- A description of any restrictions on data availability

The authors declare that all the data supporting the findings of this study are available within the paper and its supplementary information files. Sequence data of original plasmids were deposited in GenBank and original plasmids are available upon request for research and teaching purposes. All vector information is provided in Supplementary Table 1. Synthetic guide RNA target sequences are provided in Supplementary Table 2 and qPCR primer sequences in Supplementary table 4 . Numerical data and detailed statistical analysis for each figure is provided in Supplementary Table 5.

Field-specific reporting

Please select the one below that is the best fit for your research. If you are not sure, read the appropriate sections before making your selection.

[x] Life sciences [ ] Behavioural & social sciences [ ] Ecological, evolutionary & environmental sciences

For a reference copy of the document with all sections, see nature.com/documents/nr-reporting-summary-flat.pdf

Life sciences study design

All studies must disclose on these points even when the disclosure is negative.

Sample size	No sample size calculation was performed. n = 3 biologically independent samples were predicted to be sufficient for detecting statistically relevant differences between compared groups in cell culture experiments.
Data exclusions	All data was included
Replication	All the experimental findings were reliably reproduced.
Randomization	No animal or human research participants were involved. For cell culture experiments no covariates based on sample allocations to experimental groups could be observed.
Blinding	No animal or human research participants were involved. For cell culture experiments investigators were not blinded.

Reporting for specific materials, systems and methods

We require information from authors about some types of materials, experimental systems and methods used in many studies. Here, indicate whether each material, system or method listed is relevant to your study. If you are not sure if a list item applies to your research, read the appropriate section before selecting a response.

Materials & experimental systems

n/a	Involved in the study	
[x]	[ ]	Antibodies
[ ]	[x]	Eukaryotic cell lines
[x]	[ ]	Palaeontology
[x]	[ ]	Animals and other organisms
[x]	[ ]	Human research participants
[x]	[ ]	Clinical data

Methods

n/a	Involved in the study	
[x]	[ ]	ChIP-seq
[x]	[ ]	Flow cytometry
[x]	[ ]	MRI-based neuroimaging

Eukaryotic cell lines

Policy information about cell lines

Cell line source(s)	HEK-293T cells were obtained from the American Type Culture Collection (ATCC): CRL-11268<br>Jurkat (Clone E61, ATCC: TIB152) was a kind gift from Sai Reddy lab (ETHZ, Switzerland).<br>hMSC-TERT was a kind gift from Moustapha Kassem lab (University Hospital of Aarhus and University Hospital of Odense, Denmark)
Authentication	HEK-293T cells were recently obtained from ATCC and phenotype was frequently controlled by microscopy.<br>Jurkat cells were examined under a microscope and by activation-dependent IL-2 expression.<br>hMSC-TERT phenotype was frequently controlled by microscopy.
Mycoplasma contamination	HEK-293T cells were recently obtained from ATCC and were not further tested for mycoplasma contamination. Jurkat and hMSC-TERT cells were not tested for mycoplasma contamination.
Commonly misidentified lines<br>(See ICLAC register)	Cell lines used in this study are not on the list of misidentified lines.
